# Reference genes validation in *Phenacoccus solenopsis* under various biotic and abiotic stress conditions

**DOI:** 10.1038/s41598-017-13925-9

**Published:** 2017-10-19

**Authors:** Surjeet Kumar Arya, Gourav Jain, Santosh Kumar Upadhyay, Sarita, Harpal Singh, Sameer Dixit, Praveen Chandra Verma

**Affiliations:** 1 CSIR-National Botanical Research Institute, Council of Scientific and Industrial Research RanaPratap Marg, Lucknow, UP India; 2 Academy of Scientific and Innovative Research (AcSIR), Anusandhan Bhawan, Room No: 310, 2-Rafi Marg, New Delhi, India; 3 Department of Botany, Panjab University, Chandigarh, 160014 India

## Abstract

Real-time PCR (RT-qPCR) expression analysis is a powerful analytical technique, but for normalization of data requires the use of stable reference genes. However, suitable reference genes are still not known in the case of *Phenacoccus solenopsis* under variable experimental treatments. The present study focused on the identification of stable housekeeping genes as a reference for analysis under different abiotic and biotic factors in *P*. *solenopsis*. We analyzed the relative expression of six commonly used candidate reference genes in different developmental stages, host-feeding assay, temperature treatments and field distribution conditions. Expression stabilities were analyzed by geNorm, NormFinder, and RefFinder. Under developmental and field distribution conditions, *β-Tubulin* was found to be most stable reference genes followed by *rpl32 and α-Tubulin*. In the case host feeding treatment conditions, *β-Tubulin* and *α-tubulin* identified to be the most stable reference genes, while in temperature stress, a combination of *α-Tubulin* and *rpl32* found to be suitable for normalizing the RT-qPCR data. Further, the above-identified genes were validated using RT-qPCR based gene expression analysis of four objective genes namely, *Myoinhibitory peptides* (*MIPs*), *Zinc_metalloprotease* (*Zn_Mp*), *fatty acid synthase* (*fas*) *and alpha-glucosidase*. Identified reference genes will facilitate gene expression studies in future under different stress treatments in *P*. *solenopsis*.

## Introduction

*Phenacoccus solenopsis* (Mealybug) is a devastating phloem sap feeding insect pest. It is now recognized as an aggressive invasive pest of various economically important crops^1^. It infests around 166 genera of plants belonging to 51 different families^1^. Being a sap-sucking pest, it excretes copious amount of honey dew which attracts ants and favours fungal growth that ultimately reduces the rate of photosynthesis. Heavy infestation causes withering and yellowing of leaves, fruits drop prematurely and may lead to the plant’s death. The control measures employed are mainly restricted to the usage of chemical pesticides, which are not much effective against the sap-sucking group of insect pests. The alternative strategies like silencing of vital genes through RNA interference^2–4^ and utilization of insecticidal proteins^5^ have been recently employed for the control of these insect pests. However, RNAi requires the information about the vital genes. There is scarcity of genome sequence information in *P*. *solenopsis* that significantly obstructs expression profiling, gene discovery, functional validation, and eventually pest management strategy. Analyzing differential gene expression of various important biosynthetic pathways and other stress-responsive genes, and quantification of the variable transcripts in biological and experimental samples are the prerequisite for functional genomics studies^6^. In spite of the emergence of high throughput methods such as RNA-sequencing to quantify the transcript abundance in cells and tissues, RT-qPCR remains a reliable method of choice for many, specifically when limited number of genes to be analysed^7^. Because of its high specificity, reproducibility, and sensitivity, RT-qPCR is considered as the best method for use in various experimental conditions to interpret the gene expression data^8,9^. However, RT-qPCR requires the expression value of individual reference genes for precise normalization during gene expression analysis that depends upon several critical parameters^10^. Among the most important aspects is accurate normalization for obtaining biologically meaningful expression data^11,12^. The RT-qPCR analysis depends upon the appropriate selection of the reference gene(s) in the experimental set up which should be stably expressed across various tissue developmental stages and experimental conditions^12–15^. A good reference gene should have a ubiquitous expression, very low variance, and reasonable stability under different experimental conditions. Previous studies have shown that a single gene cannot stand stable under all biotic and abiotic stress conditions^16,17^. Therefore, it is imperative to use two or more reference genes for accurate normalization. For gene expression analysis through RT-qPCR in insects a few housekeeping genes such as *Actin* (*ACT*), *GAPDH* (*Glyceraldehyde-3-phosphate dehydrogenase*), *18S rRNA* and *EF-1a* (*Elongation factor-1a*) have been commonly used as reference genes^16,18–24^. However, several studies reported inconsistency in their expression that questioned their suitability as reference genes in RT-qPCR studies^14,25^. In fact, variable expression of housekeeping genes has also been reported depending on the organism, developmental stages, and various experimental conditions^26^. Therefore, none of the genes can be universally used as reference gene and hence it is necessary to validate the stability of reference gene under desired experimental conditions to ensure proper normalization during RT-qPCR analysis in the target species^14^. In most of the hemipteran species including *P*. *solenopsis*, many reference genes have been used without their prior validation. Studies on validation of the genes for RT-qPCR is now becoming prominent in other species but still lack for the mealybug species under different environmental conditions. As a result, considering the certainty of biotic and abiotic stress related internal control genes for accurate normalization of transcripts in RT-qPCR, the present work demonstrate the expression stability of six individual reference genes namely *Actin* (*Act*), *Ribosomal protein* (*rpl32*), *β-tubulin*, *α-tubulin*, *Glyceraldehyde-3-phosphate dehydrogenase* (*GAPDH*) and *Succinate dehydrogenase* (*SDH*) in *P*. *solenopsis* is subjected to individual biotic and abiotic stress treatments. Further, the identified genes were validated using RT-qPCR based gene expression analysis of three objective genes namely, *MIPs*, *Zn_MPs* and *fatty acid Synthase* and alpha- glucosidase.

## Results

### Selection of candidate reference genes, sample size, primer specificity and amplification efficiency

A total of six potential candidate reference genes namely, *Actin* (*ACT*), *ribosomal protein L32* (*rpl32*), *Glyceraldehyde-3-phosphate dehydrogenase* (*GAPDH*), *β-Tubulin*, *α-Tubulin*, and *Succinate dehydrogenase* (*SDH*) were selected for gene expression analysis using RT-qPCR in *P*. *solenopsis*.

Samples were taken into two biotic conditions (Sub group: developmental stages and field distribution) and two abiotic condition (Sub group: temperature variations and host feeding treatment). Further in each sub group, two variable conditions were taken as described in material and method section, and the detailed flow-chart of the experiment performed is shown in supplementary Fig. S1 (Total sample size, n = 48). RNA was isolated from all samples separately and treated with DNaseI to remove DNA contamination (Supplementary Fig. S2), Further PCR reaction carrying DNaseI treated RNA as a template does not showed any amplification while desired band size was obtained in PCR (Used cDNA as template), further ruled out any possibility of DNA contamination (Supplementary Fig. S2).

**Figure 1:**
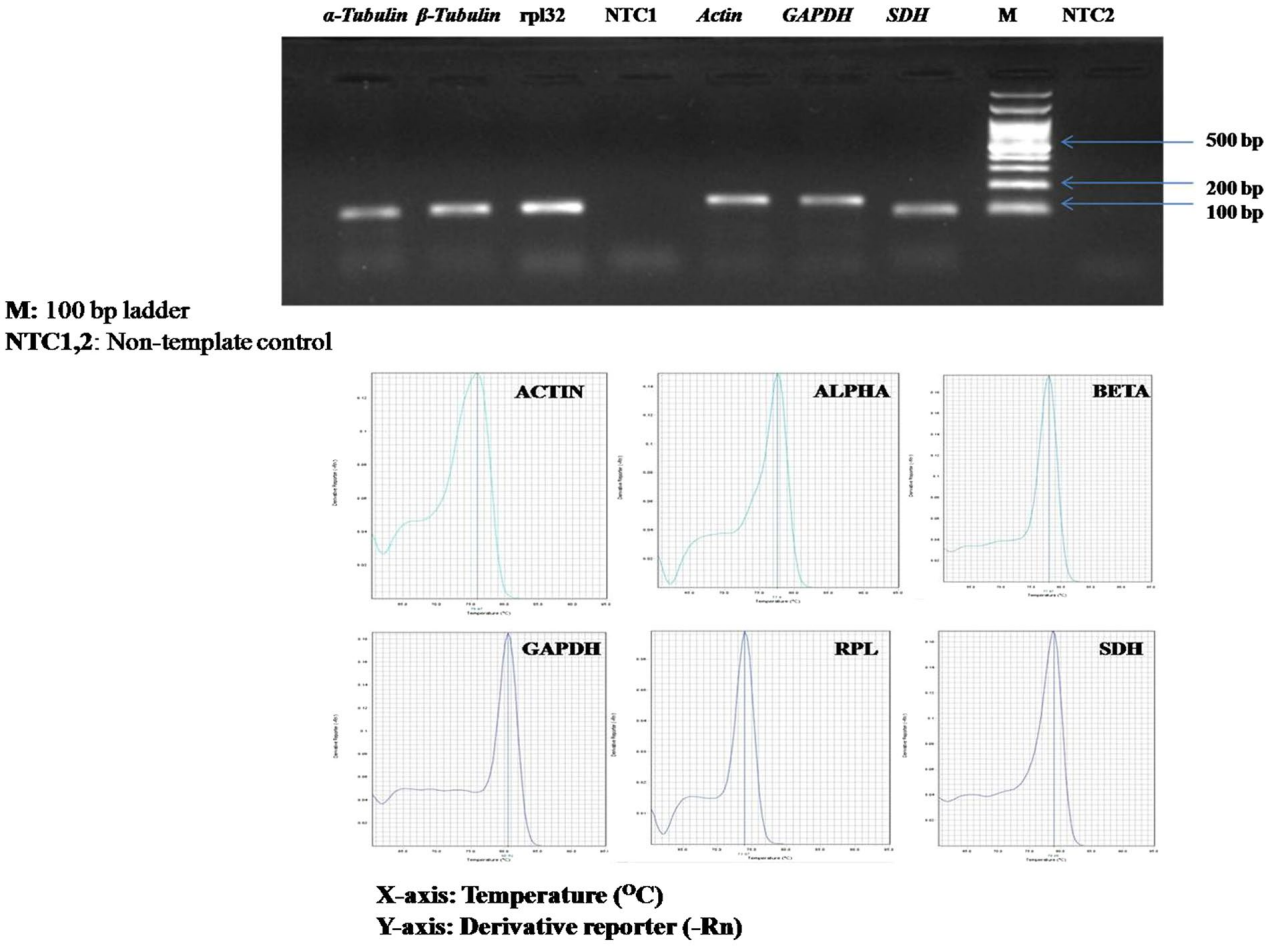
Specificity of qRT-PCR amplicons, (**a**) 1.5% agarose gel electrophoresis showing amplification of a single product of the expected size for each reference gene. ‘M’ represents 100 bp DNA Ladder. (**b**) Dissociation curves with single peaks generated from all amplicons.

RT-qPCR primers specificity was analyzed using agarose gel electrophoresis and melt curve for the individual gene. Single desired size band and amplicon peak suggested the absence of non-specific PCR products and primer-dimers (Fig. 1). The PCR Amplification efficiencies (E) for all the primer pairs were calculated using a fivefold dilution series of pooled cDNA and measured in triplicate. PCR efficiencies (E) and correlation coefficients (R2) of the six candidate reference genes were evaluated by plotting the standard curves with their slopes obtained by serial dilution that fall under allowed range of 1.9 to 2.0 for E, and 0.97–1.00 for the R2. The description of six candidate reference genes, accession numbers, primer sequences, amplicon lengths, slope, R2 and amplification efficiency are enlisted in Supplementary Table S1.

## Expression stability of the candidate reference genes

The expression profile of all six candidate reference genes was assessed by RT-qPCR, and the Ct values indicating differential transcript level were examined under four subgroups additionally all-samples subgroup (total five). The mean Ct values of these genes ranged from 18.63 to 27.76 (Table 1). The distribution of Ct values is listed in Supplementary Table S2.

**Table 1:** Expression levels of different reference genes under study in all five experimental sets.

Gene Name	Field distribution (C_t_ ± SD)	Development stages (C_t_ ± SD)	Temperature treatments (C_t_ ± SD)	Different host (C_t_ ± SD)	All samples (C_t_ ± SD)
*α-Tubulin*	16.27 ± 0.33	17.6 ± 0.13	35.57 ± 0.31	16.31 ± 0.23	21.44 ± 9.44
*β-Tubulin*	22.08 ± 0.52	21.97 ± 0.06	22.04 ± 0.28	22.11 ± 0.48	22.05 ± 0.06
*ACT*	18.68 ± 0.39	17.91 ± 0.08	19.33 ± 0.45	18.61 ± 0.20	18.63 ± 0.58
*Rpl32*	20.06 ± 0.43	18.38 ± 0.13	19.02 ± 0.25	19.52 ± 0.31	19.24 ± 0.72
*GAPDH*	19.35 ± 0.25	18.56 ± 0.18	19.25 ± 0.71	19.30 ± 0.26	19.11 ± 0.37
*SDH*	25.12 ± 0.42	25.59 ± 0.36	36.23 ± 0.32	24.09 ± 0.31	27.76 ± 5.68

**Figure 2:**
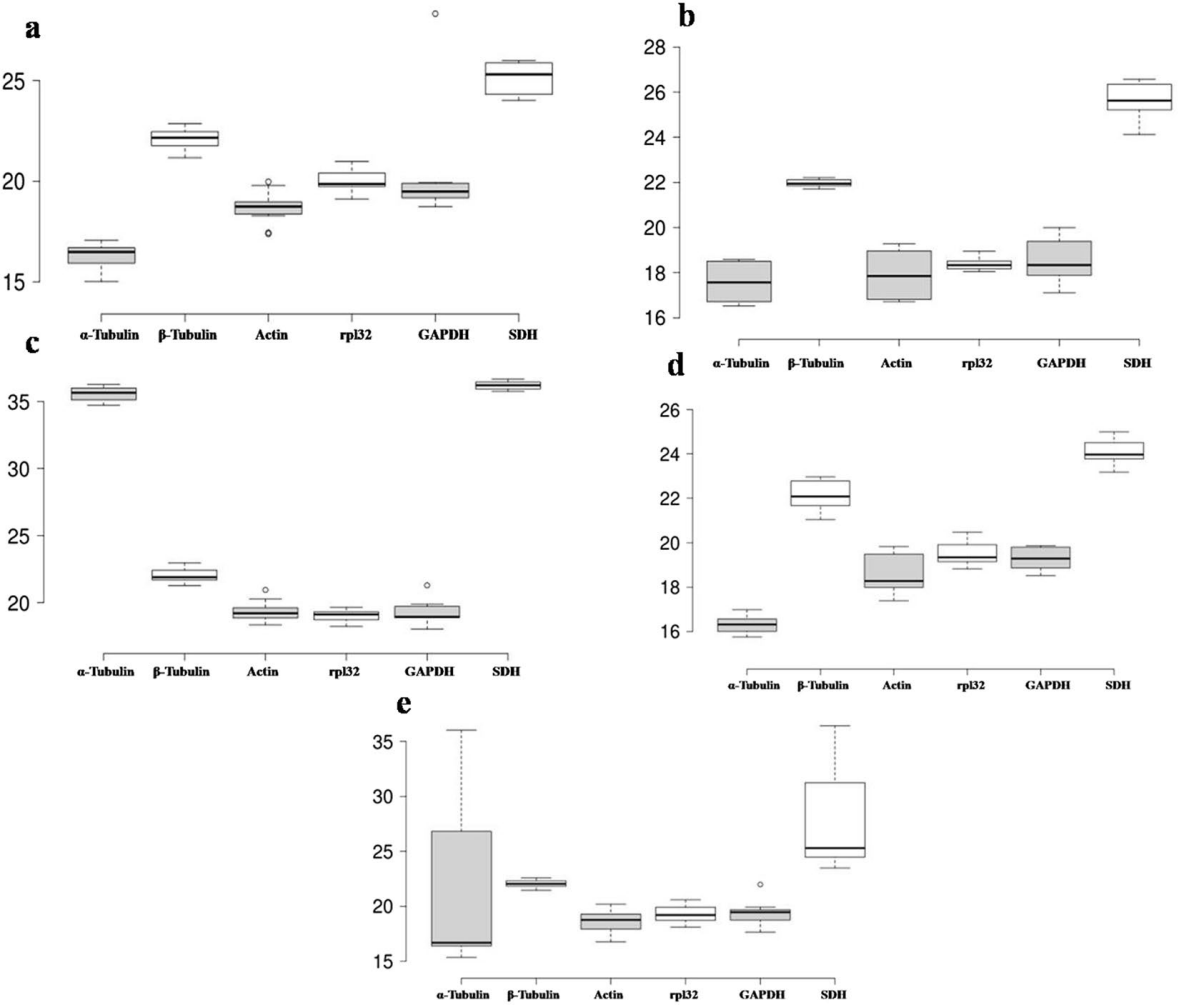
Expression range of Ct values of six candidate reference genes. (**a**) Field distribution, n = 12 sample points (**b**) Developmental stages, n = 12 sample points and (**c**) Abiotic stress-temperature treatments, n = 12 sample points (**d**) Different Host types, n = 12 sample points. (**e**) All Samples group, n = 16 sample Points. Black line across each box represents the median. Box limits indicate the 25th and 75th percentiles as determined by R software; whiskers extend 1.5 times the inter-quartile range from the 25th and 75th percentiles, outliers are represented by dots. Ct values and genes are shown on Y- and X-axis, respectively.

In all-samples subgroup, the mean Ct values ranged from a minimum of 18.63 ± 0.58 to the maximum of 27.76 ± 5.68 with highest and lowest expression levels for *ACT* and *SDH*, respectively. In the field distribution subgroups, the mean Ct values showed a minimum of 16.27 ± 0.33, and the maximum of 25.12 ± 0.42 with highest and lowest expression levels for *α-Tubulin* and *SDH*, respectively. In developmental stages distribution mean Ct values for minimum and maximum expression were 17.60 ± 0.12 and 25.59 ± 0.36 for *α-Tubulin*, and *SDH*, respectively. *Rpl32*, *α-Tubulin* and *SDH* also showed a minimum and maximum average Ct values in abiotic stress including temperature treatments and host feeding assays sample set. The two most abundant genes were *Actin* (18.63 ± 0.58 cycles) and *GAPDH* (Ct = 19.11 ± 0.37 cycles) which showed moderate variation in Ct range of 0.91 and 0.96 cycles, respectively. Additionally, CV of the Ct values was also calculated to evaluate the expression levels of candidate reference genes under all five experimental subgroups, where higher values represent higher variability or minimum stability and vice-versa. The CV of six reference genes among all samples ranged between 1 to 2%. *α-Tubulin* was the least variable reference gene with a CV of 1.26% and *GAPDH* was the most variable with a CV of 1.81%. The stability ranking of all candidate reference genes on the basis of CV values was as follows: *GAPDH* < *β-Tubulin* < *Actin* < *rpl32* < *SDH* < *α-Tubulin* (Table 2). During gene expression profiling across the sample sets in all the samples subgroups (field distribution; Fig. 2a, developmental stages; Fig. 2b, temperature variations; Fig. 2c, and different host feeding assay; Fig. 2d) separately, genes *Actin*, *β-Tubulin*, *rpl32*, *SDH* and *GAPDH* showed significant variation in Ct value (p-value < 0.05; Mann-Whitney U Non-parametric test) in biotic stress in comparison to the abiotic stress treatments (Supplementary Fig. S3). The results indicated that none of the candidate reference genes had constant expression levels across the different sample sets of *P*. *solenopsis*. Therefore, it was necessary to validate the expression stability of various candidate reference genes.

**Table 2:** Details of C_t_ and CV values of each of the selected candidate reference genes tested in *Phenacoccus solenopsis*.

Gene Name	Description	Accession no.	C_t_ ± SD	CV
*α-Tubulin*	α-Tubulin	KJ909508	21.44 ± 9.44	1.26
*β-Tubulin*	β-Tubulin	KJ909511	22.05 ± 0.06	1.52
*ACT*	Actin	KF384511	18.63 ± 0.58	1.49
*Rpl32*	Ribosomal Protein L32	KJ909510	19.24 ± 0.72	1.44
*GAPDH*	Glyceraldehyde-3-phosphate dehydrogenase (GAPDH)	KJ909509	19.11 ± 0.37	1.81
*SDH*	Succinate dehydrogenase (SDH)	KM098145	27.76 ± 5.68	1.31

## Expression stability ranking of candidate reference gene

Expression stability of the candidate reference genes was evaluated by geNorm (expression stability values M), NormFinder and RefFinder which calculated the stability values of each reference gene using the Ct values across all the experimental sets (Tables 3 and 4). The stability ranking analyses carried out by each of the three algorithms is described in the following sections.

**Table 3:** Expression stability ranks of six candidate reference genes in biotic stress treatments calculated using geNorm (GN), NormFinder (NF) and RefFinder methods.

Rank (Field Distribution)	geNorm		NormFinder		RefFinder	
	Genes	Normalization Value (MV)	Genes	Stability Value (SV)	Genes	Geomean Of Ranking Value
1	*β-Tubulin*	0.38	*rpl32*	0.13	*β-Tubulin*	1
2	*rpl32*	0.42	*β-Tubulin*	0.17	*rpl32*	1.86
3	*α-Tubulin*	0.55	*α-Tubulin*	0.35	*α-Tubulin*	3.22
4	*SDH*	0.65	*SDH*	0.43	*GAPDH*	3.76
5	*ACT*	1.07	*ACT*	0.47	*SDH*	4.68
6	*GAPDH*	1.18	*GAPDH*	0.55	*ACT*	5.73
**Rank (Developmental stages)**	**geNorm**		**NormFinder**		**RefFinder**	
	**Genes**	**Normalization Value (MV)**	**Genes**	**Stability Value (SV)**	**Genes**	**Geomean Of Ranking Value**
1	*β-Tubulin*	0.55	*rpl32*	0.16	*β-Tubulin*	1.19
2	*rpl32*	0.65	*β-Tubulin*	0.17	*rpl32*	1.41
3	*α-Tubulin*	0.87	*α-Tubulin*	0.70	*SDH*	3.46
4	*SDH*	0.94	*GAPDH*	0.77	*GAPDH*	3.66
5	*ACT*	1.39	*ACT*	0.77	*α-Tubulin*	4.73
6	*GAPDH*	1.48	*SDH*	0.78	*ACT*	6

**Table 4:** Expression stability ranks of six candidate reference genes in abiotic stress treatments and all sample groups calculated using geNorm (GN), NormFinder (NF) and RefFinder methods.

Rank (Different temperature variation)	geNorm		NormFinder		RefFinder	
	Genes	Normalization Value (MV)	Genes	Stability Value (SV)	Genes	Geomean Of Ranking Value
1	*GAPDH*	0.33	*rpl32*	0.02	*rpl32*	1.19
2	*β-Tubulin*	0.34	*α-Tubulin*	0.08	*α-Tubulin*	2.28
3	*α-Tubulin*	0.49	*SDH*	0.19	*SDH*	2.63
4	*rpl32*	0.52	*GAPDH*	0.21	*β-Tubulin*	2.99
5	*SDH*	0.52	*β-Tubulin*	0.22	*ACT*	4.73
6	*ACT*	0.61	*ACT*	0.23	*GAPDH*	6
**Rank (Different host feeding assays)**	**geNorm**		**NormFinder**		**RefFinder**	
	**Genes**	**Normalization Value (MV)**	**Genes**	**Stability Value (SV)**	**Genes**	**Geomean Of Ranking Value**
1	*β-Tubulin*	0.34	*rpl32*	0.24	*GAPDH*	1.19
2	*α-Tubulin*	0.34	*α-Tubulin*	0.37	*rpl32*	2
3	*SDH*	0.46	*SDH*	0.42	*α-Tubulin*	2.83
4	*rpl32*	0.68	*β-Tubulin*	0.44	*SDH*	3
5	*GAPDH*	0.75	*GAPDH*	0.56	*β-Tubulin*	5
6	*ACT*	1.03	*ACT*	0.63	*ACT*	6
	**geNorm**		**NormFinder**		**RefFinder**	
**Rank (All sample groups)**	**Genes**	**Normalization Value (MV)**	**Genes**	**Stability Value (SV)**	**Genes**	**Geomean Of Ranking Value**
1	*α-Tubulin*	0.24	*rpl32*	0.08	*rpl32*	2
2	*ACT*	0.29	*β-Tubulin*	0.16	*ACT*	2
3	*SDH*	0.36	*SDH*	0.21	*β-Tubulin*	2.06
4	*rpl32*	0.45	*GAPDH*	0.22	*GAPDH*	2.45
5	*β-Tubulin*	0.47	*α-Tubulin*	0.35	*SDH*	5
6	*GAPDH*	0.77	*ACT*	0.49	*α-Tubulin*	6

### geNorm analysis

**Figure 3:**
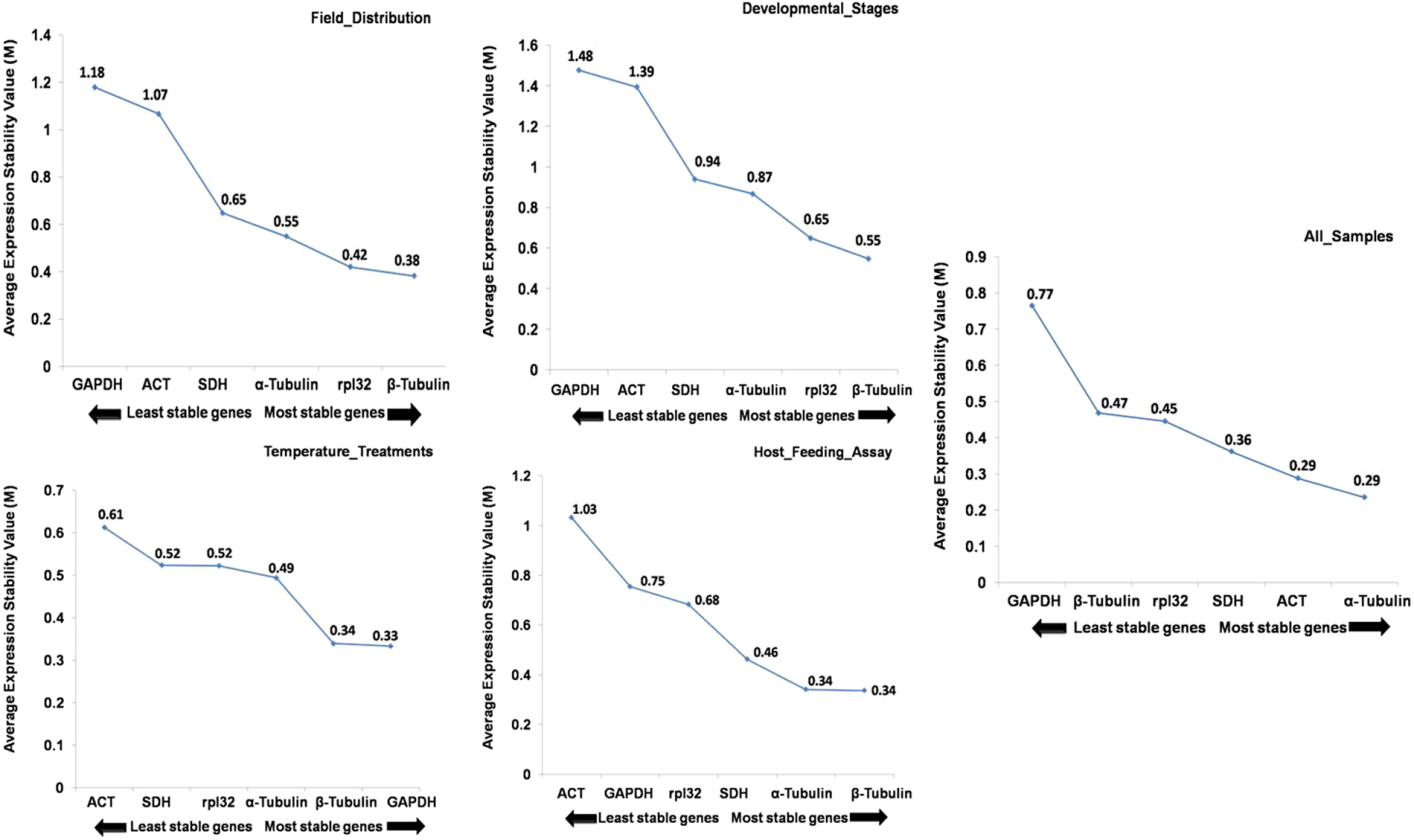
Gene expression stability and rankings of six candidate reference genes using Expression stability values (M). The average expression stability (M) was calculated following stepwise exclusion of the least stable gene across all the samples within an experimental set, the lowest M value indicates the most stable gene, while the highest value represents the most variable gene.

The gene expression stability values of all the genes were calculated using the geNorm statistical algorithm. Expression stability of individual gene was referred by the M-Value which is computed as the standard deviation of the log-transformed expression ratios across samples for the particular gene in relation to other reference genes in the genes panel^27^. The calculation involved stepwise exclusion of particular gene with the highest M Values (i.e. the least stable gene) from the panel until reaching the last two genes with the smallest M values (i.e. the most stable genes). As per calculation, the gene with the lowest M value was considered as the most stable, while the M values > 1.5 were considered as unacceptable levels of expression variability. To determine the suitable stable reference genes under specific experimental conditions, the genes were ranked from the most stable to the least stable in each sample set. During biotic and abiotic stress conditions, each gene showed the significant expression stability with the threshold value below 1.5. In all the sample subgroup set, *α-Tubulin* and *ACT* had least M value (0.24 and 0.29), followed by *SDH* and *rpl32* (0.36 and 0.45). However, *GAPDH* exhibited highest M value (0.77). The results indicated that *α-Tubulin* and *ACT* were most stable in expression, while *GAPDH* was the least stable. In field distribution set, *GAPDH* (M = 1.18) was the least stable gene and *β-Tubulin* and *rpl32* (M = 0.38 and 0.42) were identified as the best pair of reference genes. Similarly, *GAPDH* (M = 1.48), and *ACT* (M = 1.39) was the least stable during developmental stages, while *β-Tubulin* (M = 0.55) and *rpl32* (M = 0.65) showed stable expression. During different host feeding assays, *β-Tubulin* (M = 0.34) and *α-Tubulin* (M = 0.34) were identified as the best-ranked reference genes, whereas *ACT* was the least stable gene (M = 1.03). During temperature variations, *β-Tubulin* (M = 0.34) and *GAPDH* (M = 0.33) were identified as the best pair of reference genes, while *ACT* and *SDH* (M = 0.61 and 0.52) were considered as the least stable genes (Fig. 3; Table 3 and Supplementary Table S3).

## Optimal number of internal candidate genes for normalization

**Figure 4:**
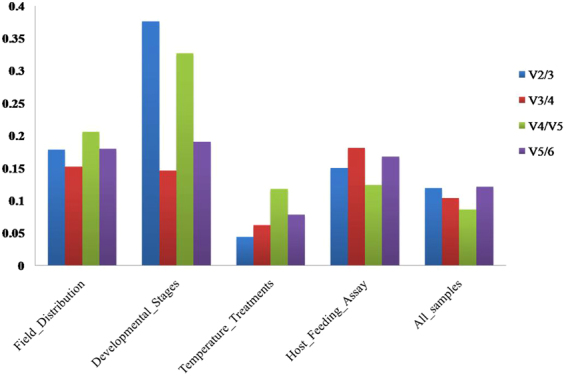
Determination of the optimal number of reference genes for geNorm analysis. The pairwise variation (Vn/Vn + 1) was analyzed for the normalization factors NFn and NFn + 1 by geNorm program to determine (V < 0.15) the optimal number of reference genes.

To produce accurate and consistent results, two or more genes are required for the RT-qPCR experiment. To obtain the optimal number of reference genes for data normalization, we calculated the pairwise variation (Vn/n + 1) of serial log-transformed NF ratios using N relative to N + 1 reference genes (i.e. log2 (NFn/NFn + 1) as previously described^27^. Since the individual reference gene had considerably differential expression across the samples, NF could be sensitive to the stepwise inclusion of these reference genes resulting in an increase or decrease in Vn/n + 1 value. A cut-off value of V = 0.15 was recommended in deciding the addition of the next reference gene. A large pairwise variation with a significant effect means that the added gene is preferably included for calculation of a reliable normalization factor. In contrast, an extra reference gene is not necessary for normalization when the cut-off value is below 0.15. As shown in Fig. 4, the Pairwise variations of all biotic treatment sample set, V2/3 value were above the cut- off value 0.15, which indicates that two reference genes were not enough for accurate normalization. The addition of the third or fourth reference gene was required for significant normalization of the results. In our study, the V2/3 values of the developmental set of group were above the proposed 0.15 cut-off. But V3/4 values were below the cut-off and these results indicate that normalization with three (*α-Tubulin*, *rpl32* and *β-Tubulin*) stable reference genes was required to produce valid expression data set. In all samples sets, each Pairwise variation values were below the proposed 0.15 cut-off. In the case of field distribution, the V3/4 was close to the proposed 0.15 cut-off. Moreover, the inclusion of additional reference genes did not lower the V value below the recommended 0.15 cut-off until the fourth or five genes was added. In temperature treatments, the V2/3 values were below the proposed 0.15 cut-off value (Fig. 4). According to geNorm, two reference genes (*β-Tubulin* and *GAPDH*) should be required for a suitable normalization in the high or low temperature-stressed samples. In the experimental set of host feeding assay, all the pair-wise variation V2/3 values were below the recommended 0.15 cut-off. These results indicate that normalization with additional reference genes did not lower the V value below the proposed 0.15 cut-off.

### NormFinder analysis

The result of NormFinder analysis presented that the stability ranking of the six candidate reference genes was relatively consistent with the data array of geNorm findings. The results of gene expression stability analysis are shown in Supplementary Table S4. For the all samples set *rpl32*, *β-Tubulin* and *SDH w*ere identified as the best three optimal internal control genes with the stability value of 0.08, 0.16, and 0.21, while *ACT* with stability value (SV) 0.49 showed higher variation. In the case of field distribution, *ACT* and *GAPDH* were unstable with the stability value of (0.47), (0.55). But in the sample set of developmental stages, *ACT* and *SDH* showed stability value of (0.77), (0.78) and said to be very unstable. The *rpl32*, *β-Tubulin* and *α-Tubulin* found to be highly stable with the stability of 0.16, 0.17 and 0.70 in developmental conditions, respectively. The *β-Tubulin* and *rpl32* were also very stable and showed the stability value of (0.38 and 0.42) at field distribution sample sets (Table 3). This means that *α-Tubulin*, *β-Tubulin*, and *rpl32* were the best stably expressed gene under developmental subgroups, and *β-Tublin* and *rpl32* performed better in the field conditions. In the sample set temperature variations, *ACT* and *β-Tubulin* were found to be very unstable with the stability values of (0.23 and 0.22, respectively) while *α-Tubulin* and *rpl32* behave stable with the stability of (0.08 and 0.02). Under different host feeding assays, *β-Tubulin* and *ACT* found to be very unstable with stability values of (0.44, 0.63), while *rpl32* and *GAPDH* were stable with stability value of (0.24, 0.56). The results shown with NormFinder was similar to the one obtained from geNorm to some extent (Tables 3 and 4). Overall, NormFinder predicted the reference genes *α-Tubulin*, *β-Tubulin* and *rpl32* were the highly stable genes in development and host feeding assay sample, which was set similar to the results obtained by geNorm analysis. In the case of field distribution and temperature treatments, *α-Tubulin* and *rpl32* were the highly stable genes and *ACT*, *GAPDH* were the unstable gene in the all sample set subgroups.

### RefFinder analysis

The RefFinder analysis revealed that *α-Tubulin* and *β-Tubulin* were most stable genes in field distribution samples subgroup. However, *β-Tubulin* and *rpl32* were ranked as top in different stages sample subset. The *rpl32*, and *α-Tubulin* genes were the most stable genes under different temperature conditions, whereas *GAPDH*, *rpl32* and *α-Tubulin* were observed to be highly stable in different host feeding assays. The comprehensive ranking also revealed that the *β-Tubulin* and *rpl32* were the most stable genes in the all biotic stress treatment samples, while *rpl32* and *α-Tubulin* in abiotic stress treatments. Other candidate genes such as *ACT* in the field, different stages and host feeding assay sample subsets; and *GAPDH* in temperature treatments were found as the unstable genes in this analysis (Supplementary Table S5).

### Expression analysis of *MIP*, *Zn_Mp*, alpha-glucosidase and fatty acid synthase gene from *P*. *solenopsis* under different biotic and abiotic stress conditions for reference genes validation

In order to validate the selected reference gene, the differential expression of four objective genes namely *Myoinhibitory peptide* (*MIP*; for the developmental stage and host feeding), *Zinc_metalloprotease* (*Zn_Mp*; for developmental stage), fatty *acid synthase* (*fas*; for temperature stress) and *alpha-glucosidase* (for field distribution) were analyzed by RT-qPCR.

**Figure 5:**
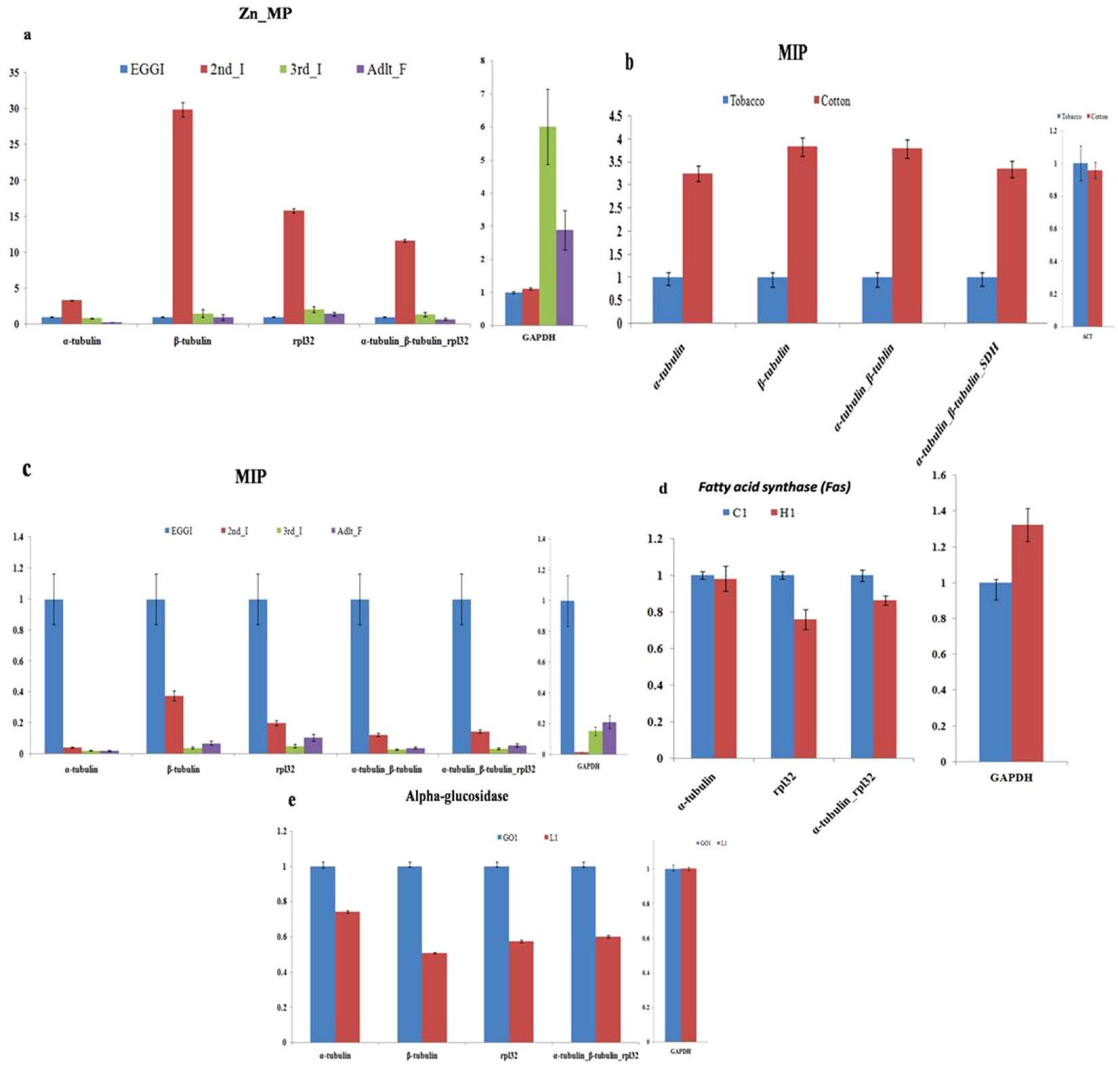
Variation in *Zn_MP*, *MIP*, fatty acid synthase and alpha glucosidase gene-expression data normalized by different reference genes and their combinations. (**a**) *Zn_MP* expression levels in developmental stages (**b**) *MIP* expression levels in Host-Feeding Assay’s and developmental stages. (**c**) Fatty acid synthase expression levels in different temperature treatment conditions. (**d**) Alpha glucosidase expression levels in field treatment conditions. Bar indicate the standard error (±SE) evaluated from three biological replicates.

The expression profiles of the genes *MIP* and *Zn_Mp* were normalized using the combination of most stable reference gene (*β-tubulin*, *α-tubulin*, and *rpl32*) and the least stable reference gene *GAPDH* and *ACT* recommended by geNorm, RefFinder, and NormFinder. Relative transcript accumulation of *MIP* was found to be unbiased when *α-Tubulin*, *β-Tubulin*, *rpl32* or combination of *α-Tubulin*_*β-tubulin*_*rpl32* were used confirming the high level of expression stability of these three genes. Using *α-Tubulin*, *β-Tubulin*, *rpl32* or combination of *it*, the expression of *MIP* and *Zn_Mp* were increased under different developmental and host feeding treatment conditions (Fig. 5a,b). For field distribution and temperature treatment conditions, expression profiles of *alpha glucosidase* and *Fas* were normalized using the most stable reference genes *α-Tubulin*, *β-Tubulin* and *rpl32* and combination of it (Fig. 5c,d). *Fas*, which plays a central role in lipid synthesis and in stress tolerance showed increase in their relative expression during any abiotic stress conditions^28^. The alpha-glucosidase has role in the metabolism of oligosaccharides and play major role in carbon nutrition and osmoregulation^29^. The relative expression pattern of *MIP*, *Zn_Mp*, *alpha-glucosidase* and *Fas* showed strong deviation and failed to achieve consistency when *GAPDH* and *ACT* for transcript normalization under development, host feeding treatments, field distribution and different temperature treatments.

## Discussion

Gene expression analyses during different developmental stages and environmental conditions are extremely important to reveal the molecular mechanisms underlying various biological processes. RT-qPCR is the best technique even for low abundant mRNA transcripts and is becoming one of the most commonly utilized technology for the analysis of gene expression in recent years^7,13,26,30–33^. However, accurate normalization of gene expression remains a major concern for precise RT-qPCR analysis. The normalization of gene expression value reduces the variations occurred during sample preparations, various steps of RT-qPCR, cDNA preparations, PCR efficiency, and various others. The majority of studies used one gene for their data normalization which produces an ambiguous result, and the gene was often selected on the basis of earlier reports rather than on their suitability for a particular experimental conditions. Utilization of right reference genes is a key strategy to minimize the variations in expression analysis. Earlier, it has been demonstrated the use of two or more reference genes during RT-qPCR provides more precise results^27^. In this context, there is an utmost need of suitable reference genes for expression analysis in economically devastating pest of cotton, *P*. *solenopsis* and other hemipteran pest of agricultural crops as well.

The PCR amplification efficiency is directly related to master mix performance, sample quality and assay performed. Our result suggested, the amplification efficiencies and correlation coefficients of the six candidate reference genes were ranges of 1.9 to 2.0, and 0.97–1.00, respectively, which is similar to the previous reports^34^. Numerous statistical algorithms such as geNorm^27^, NormFinder^11^, RefFinder (http://fulxie.0fees.us/?type=reference), BestKeeper^35^ and delta Ct^36^ have been developed to estimate the expression stability of individual reference genes, but the rankings of the best reference genes were different with the different programs. So, for accurate normalization in gene expression studies, a comparison of different algorithms allows better evaluation of gene expression patterns^37^. We found that the MV value of all analyzed six reference genes were less than 1.5 under different abiotic and biotic stress conditions, this indicating possible expression stability of selected reference genes^27^. All three of the algorithms (geNorm, NormFinder, and RefFinder) used in present study recommended *α-tubulin*, *β-tubulin* and *rpl32* as the top three most stable reference genes in all the samples set across all the treatment conditions with some variability in their ranking order. *α-Tubulin*, β*-Tubulin* constituted the most stable reference gene pair with *rpl32* according to geNorm in the biotic stress treatment conditions. We consistently found *α-Tubulin*, *β-Tubulin* as best reference genes this may be due to their structural requirement of the cell so their expression could be consistent in organism invariable to any treatment. Under developmental, *β- Tubulin* was found to be most stable reference genes followed by *rpl32* and *α-Tubulin*. An earlier study on *Frankliniella occidentalis* suggested *EF-1* and *rpl32* are the most stable reference genes in different developmental stages^38^. Although *rpl32* is common in both studies but we cannot compare these result simultaneously due to the difference in model insect and experimental result analysis. Further, *α_tubulin* and *rpl32* are the best reference genes under different temperature conditions; these results are consistent with the earlier finding where *α_tubulin* and *rpl32* selected as best reference genes under heat stress in *P*. *solenopsis* ^39^. In field distribution, *ACT* and *GAPDH* were used as the stable gene to normalize the data in *Aphis gossypii*, but we found both genes were unstable in expression. This might be due to change in different climatic conditions of collection field^34^.

The applicability of the reported three most stable genes were assessed by analyzing their expression pattern of *MIP*, *Zn_MP*, *alpha-glucosidase* and *fas* under different biotic and abiotic stress conditions. The objective genes showed amplification with three of the internal control genes *α-Tubulin*, *β-Tubulin*, and *rpl32* under different biotic and abiotic stress. However, they showed variations when gene expression was assessed by using the unstable genes, *ACT*, and *GAPDH*. The results showed that the expression stability of the six most commonly used reference genes in *P*. *solenopsis* was varied. Of these six reference genes; *α-Tublin*, *β-Tublin*, and *rpl32* showed least changes in expression level in developmental stages, field distribution and host feeding assay treatment conditions. The *ACT and GAPDH* expression were found to be most variable during biotic stress and host feeding assay. None of the single reference genes showed constant expression in all the four different experimental conditions. Reportedly, expression of each gene was profoundly affected by the different experimental conditions on the chosen reference genes, though all of them were not changed by each treatment conditions. Collectively, the results re-confirmed the previous report that no single reference gene is showing the same expression across all the treatment conditions. The best reference gene(s) under different experimental conditions were indicated by rank order. However, our results showed that *ACT* and *GAPDH* are also not an ideal reference gene for all the studies of *P*. *solenopsis*, it is only suitable under certain experimental conditions and needed to cooperate with other reference genes to obtain the reliable results. For instance, in the temperature stress experiments, the combination of *rpl32* and *α-Tubulin* could be suitable for normalizing the RT-qPCR data. In the developmental stage and field distribution condition, the use of combining the expression of three reference genes namely, *α-tublin*, *β-Tubulin* and *rpl32* gave better results. Similarly, when the combination of *α-Tubulin and β-Tubulin* were used for normalizing the analytical RT-qPCR results of host feeding assays, an accurate result could be obtained. The results demonstrated that using unsuitable reference gene(s) for normalization might lead to deviated results. Therefore, it is a perquisite to select appropriate reference genes for accurate estimation of target gene expression.

In conclusion, we have investigated the expression of six candidate reference genes at different biotic and abiotic treatment conditions, an attempt to identify most suitable reference genes for normalizing gene expression in *P*. *solenopsis*. Our results suggested that we could use combination of reference genes for the different experimental set-up to avoid any variations in gene expression to achieve perfect normalization. To the best of our knowledge, this is one of the first reports on the evaluation of candidate reference genes across different experimental conditions in *P*. *solenopsis* and will provide guidance to other researchers to select reference genes against target genes by RT-qPCR in the related species.

## Methods

### Insect rearing

*P*. *Solenopsis* was reared on cotton plants for biological studies in the laboratory at CSIR-NBRI, Lucknow, India. Cotton plants twigs infested with reproducing females of *P*. *Solenopsis* were brought to the laboratory; individual females were separated, and reared on cotton (*Gossypium Hirsutum* L.) plants under controlled conditions at 25–28 °C, 70–80% relative humidity and photoperiods of 16:8 (L: D) h. As per the experimental requirement, desired stages of insect (eggs, nymphs, and adults) were collected from plants.

### Samples collection

We collected the differential populations of mealybug encompassing a range of experimental conditions:

### Biotic factors

#### Development stages

Different developmental stages (egg, first instar nymphs, adult) of *P*. *solenopsis* were collected from reared population. Due to the lack of sufficient biological material, egg and first instar nymphs were pooled and considered as a single sample while adult insect was taken as separate sample (Total two samples). Further samples were collected from two different plant considers as biological duplicate, while the experiment was performed in triplicate with each sample.

### Field distribution

A field variation has a great impact on the growth parameters of the insect due to change in temperature and relative humidity at the place of maintenance of the insect populations and could be used as one of the factors for gene expression studies. Two geographical populations were collected from two physically separated fields (~800 Km) one from the cotton field of CSIR-NBRI, Lucknow (26°55′N, 80°59′E) and the other from Panjab University, Chandigarh (30.7601°N, 76.7663°E). Collected populations were multiplied in the net-house conditions and glass house conditions, separately. Hundreds of adult stage female populations were collected separately and stored in RNAse free micro-centrifuge tubes containing RNA later solution and stored in −80 °C until use.

### Abiotic factors

#### Temperature treatments

Temperature has a great impact on the insect developmental cycles. To look out for variability in the growth parameters with a varied range of temperatures, adult mealybugs were exposed to two temperature regimes 15 °C and 42 °C for 1 h. In total, a hundred adults at each temperature points were collected for RNA extraction.

### Host plant-feeding assays

Feeding assay was designed to evaluate the growth of adult mealybugs on cotton and tobacco leaves. Leaves of both plants were collected from the glass house and washed thoroughly and dry on bloating sheet. Leaves were cut and placed in petridish (35 mm) pre-poured with 1% agarose. Adult female mealybugs were starved for 5 hours and released on a plate (5/dish). After 48 h, until it started secreting the honeydews, insects were gently brushed from leaves disc and stored in RNA later solution and stored in −80 °C until use.

### Choice of candidate reference genes and primer design

Sequences of six reference genes namely *Actin* (*ACT*), *Ribosomal Protein* (rpl32), *β-Tubulin*, *α-Tubulin*, *Glyceraldehyde-3-phosphate dehydrogenase* (*GAPDH*) and *Succinate dehydrogenase* (*SDH*) were retrieved from respective *P*. *solenopsis* partial mRNA sequences available at NCBI database (Supplementary Table 1) for subsequent RT-qPCR-primer designing and amplification. Primer pairs used in PCR reactions were designed from the non-conserved regions of the gene sequences using Primer Express 3.0 (Applied Biosystems, USA) with the following parameters: 18 ± 5 mers primer length; 100–150 bp amplicon size; 59 ± 2 °C melting temperature (Tm); 50 ± 5% guanine-cytosine (GC) content including no hairpin structures, self-complementarities or self- dimers at the 3′-end.

### Extraction of total RNA and cDNA synthesis

Total RNA was extracted using 500 μL of TRIzol^®^ Reagent (Sigma-Aldrich, St. Louis, USA) from pooled sample of Eggs and first instars nymph, and third instar and adult female mealybugs according to the manufacturer instructions. The extracted RNA was resuspended in 50 μL of Nuclease-free water (Life Technologies). RNA concentration of each sample was determined using NanoDrop-1000 spectrophotometer (NanoDrop Technologies). The OD260/OD280 nm absorption ratio (1.98–2.01) and OD260/OD230 (≥2.0), was used to determine the quality and purity of RNA preparations. RNA integrity was analyzed by means of electrophoresis in 1.5% agarose gels visualize after ethidium bromide staining using Bio-Rad Gel Doc systems (Bio-Rad, USA) (Supplementary Fig. 1). Genomic DNA was eliminated using the Turbo-DNase kit (Ambion, USA) (Supplementary Fig. 1). Further, the presence of DNA contamination was checked through PCR using DNase-treated RNA samples with −RT and +RT reaction. Minus-reverse transcriptase (“−RT”) control is a mock reverse transcription containing all the RT-PCR reagents (no cDNA template), except the reverse transcriptase and +RT samples contained all RT-PCR reagents (cDNA template). RNA samples (1 µg) were immediately used to synthesize cDNA using the SuperScript III Reverse Transcriptase (Ambion, USA) and 10 μM Oligo (dT) primers in a final volume of 20 μL. The reverse transcription reactions were performed in a Thermal Cycler (Bio-Rad, USA) under the following conditions: 5 min at 65 °C; 5 min at 25 °C; 60 min at 50 °C; and 15 min at 70 °C. Finally, cDNAs were stored at −20 °C.

### PCR and RT-qPCR

Amplification specificity of 6 primer pairs was confirmed by PCR. The PCR cocktail was having the volume of 10 μl contained 10× PCR buffer (ThermoFischer Scientific, USA), 10 mM dNTPs mix (ThermoFischer scientific, USA), 5 μM each of forward and reverse primers, 1 μl of cDNA, and 0.2 μl of Taq polymerase. The PCR program for amplification was as follows: an initial denaturation step of 3 min at 94 °C, and 30 cycles of 30 s at 94 °C, 45 sec at 60 °C and 45 sec at 72 °C followed by an extension step of 10 min at 72 °C. PCR products were separated on 1.5% agarose gels. The RT-qPCR was executed using SYBR Green detection chemistry on the 7500 fast Real-time PCR system v2.0.6 (Applied Biosystems). The reaction mixture contained 1 μl of 5 times diluted cDNA (equal to 50 ng of initial amount of RNA), 5 pico moles of each gene-specific forward and reverse primer, and 5 μl of 2 × FAST SYBR Green PCR Master Mix (Applied Biosystems, USA) in 10 μl of total reaction volume. The RT-qPCR cycling conditions were as follows: an initial denaturation step of 20 s at 50 °C, 10 min at 95 °C, and 40 cycles of 15 s at 95 °C, and 1 min at 60 °C followed by melt curve analysis using default parameters in order to check the PCR specificity by steady increase in temperature from 60 °C to 90 °C. The PCR efficiency of each primer set was determined through slope of the amplification curve in the exponential phase, obtained by five-fold dilution series of cDNA (1:5, 1:25, 1:125, 1:625, and 1:3125) using E = 10 (−1/slope)−1 (3.3 ≥ slope ≥ 3.1) by the software itself (Applied Biosystems). Triplicates of two biological replicates of each sample were used for real-time PCR analysis and three technical replicates were analyzed for each biological sample.

### Data analysis for expression stability of candidate reference genes

The candidate reference genes expression levels were determined by threshold cycles (Ct). The stability and suitability of these genes were evaluated using three independent Excel based calculations and algorithms, i.e., geNorm^27^, NormFinder^11^, and RefFinder (http://fulxie.0fees.us/?type=reference). The Ct values were imported into the Excel sheet, and average expression stability value-M (AESV-M) for each candidate gene was calculated. Then, pair-wise variation (V) of each gene was calculated and compared with all other candidate reference genes. The geNorm stability value provides brief ideas about the expression stability of the genes on the basis of stability value, M. Through the NormFinder analysis, first ct values were converted into relative quantities and ranked the stability of the reference genes depending on the variation in gene expression. Genes with the lower stability values are considered to be most stable gene and are best to select as reference gene for that particular experimental conditions. RefFinder is a web-based (http://fulxie.0fees.us/?type=reference) online tool, which integrates the presently available four advanced computational programs geNorm^27^, NormFinder^11^, comparative ΔCt method^36^ and BestKeeper^35,38^ and calculates the geometric mean for the comprehensive ranking.

### Validation of reference genes

On the basis of available literature^28,29,40–44^ and in-house transcriptome data (unpublished) four genes namely *Myoinhibitory peptide* (*MIP*), *Zinc_metalloprotease* (*Zn_Mp*), *fatty acid synthase* (*Fas*) and *alpha-glucosidase* were selected for validating three most stable reference gene *α-Tubulin*, *β-Tubulin*, and *rpl32*; and the two most varying reference genes namely *GAPDH* and *ACT* identified from this study. Sequence of the four objective genes was obtained from a *P*. *solenopsis* transcriptome *in-house* data (data not published yet) and real time primers were synthesized (supplementary Table 6). RT-qPCR was performed as described above. The relative mRNA expression data of objective genes normalized separately with internal control genes used was analyzed using 2^−ΔΔCt^ method^45^.

## Electronic supplementary material

Supplementary File

## References

[1] Nagrare, V. S. *et al*. Compendium of cotton mealybugs. *Central Institute for Cotton Research*. Nagpur, **42** (2011).

[2] Upadhyay SK (2011). RNA interference for the control of whiteflies (*Bemisia tabaci*) by oral route. Journal of biosciences..

[3] Upadhyay SK (2013). siRNA machinery in whitefly (*Bemisia tabaci*). PloS one..

[4] Thakur N (2014). Enhanced whitefly resistance in transgenic tobacco plants expressing double stranded RNA of v-ATPase A gene. PloS one..

[5] Shukla AK (2016). Expression of an insecticidal fern protein in cotton protects against whitefly. Nature Biotechnology..

[6] Kumar K, Muthamilarasan M, Prasad M (2013). Reference genes for quantitative real-time PCR analysis in the model plant foxtail millet (*Setariaitalica L*.) subjected to abiotic stress conditions. Plant Cell, Tissue and Organ Culture (PCTOC)..

[7] Nolan T, Hands RE, Bustin SA (2006). Quantification of mRNA using real-time RT-PCR. Nature protocols..

[8] Valasek MA, Repa JJ (2005). The power of real-time PCR. Advances in physiology education..

[9] Park Y (2008). Evaluation of multiplex PCR assay using dual priming oligonucleotide system for detection mutation in the Duchenne muscular dystrophy gene. The Korean journal of laboratory medicine..

[10] Majerowicz D (2011). Looking for reference genes for real‐time quantitative PCR experiments in *Rhodnius prolixus* (Hemiptera: Reduviidae). Insect molecular biology..

[11] Andersen CL, Jensen JL, Ørntoft TF (2004). Normalization of real-time quantitative reverse transcription-PCR data: a model-based variance estimation approach to identify genes suited for normalization, applied to bladder and colon cancer data sets. Cancer research..

[12] Crismani W (2006). Microarray expression analysis of meiosis and microsporogenesis in hexaploid bread wheat. BMC genomics..

[13] Huggett, J., Dheda, K., Bustin, S. & Zumla, A. Real-time RT-PCR normalisation; strategies and considerations. *Genes and immunity*. 6(4), 279–284. 10.1038/sj.gene.6364190 15815687

[14] Kozera B, Rapacz M (2013). Reference genes in real-time PCR. Journal of applied genetics..

[15] Bustin SA (2002). Quantification of mRNA using real-time reverse transcription PCR (RT-PCR): trends and problems. Journal of molecular endocrinology..

[16] Lu Y (2013). Identification and validation of reference genes for gene expression analysis using quantitative PCR in *Spodoptera litura* (Lepidoptera: Noctuidae). PloS one..

[17] Zhang S (2015). Identification and validation of reference genes for normalization of gene expression analysis using qRT-PCR in *Helicoverpa armigera* (Lepidoptera: Noctuidae). Gene..

[18] Bhatia V, Bhattacharya R, Uniyal PL, Singh R, Niranjan RS (2012). Host generated siRNAs attenuate expression of serine protease gene in *Myzus persicae*. PloS one..

[19] Li R (2013). Reference gene selection for qRT-PCR analysis in the sweetpotato whitefly, *Bemisia tabaci* (Hemiptera: Aleyrodidae). PLoS One..

[20] Puinean AM (2010). Amplification of a cytochrome P450 gene is associated with resistance to neonicotinoid insecticides in the aphid *Myzus persicae*. PLoS Genet..

[21] Zhang P (2011). Suppression of jasmonic acid-dependent defense in cotton plant by the mealybug *Phenacoccus solenopsis*. PLoS One..

[22] Upadhyay SK (2015). Whitefly genome expression reveals host-symbiont interaction in amino acid biosynthesis. PloS one..

[23] Zhang, P. J., Huang, F., Zhang, J. M., Wei, J. N. & Lu, Y. B. The mealybug *Phenacoccus solenopsis* suppresses plant defense responses by manipulating JA-SA crosstalk. *Scientific reports*. **5** (2015). 10.1038/srep09354 PMC4366759 25790868

[24] Li X (2016). Molecular Characterization of Two Fatty Acyl-CoA Reductase Genes From *Phenacoccus solenopsis* (Hemiptera: Pseudococcidae). Journal of Insect Science..

[25] Gutierrez L, Mauriat M, Pelloux J, Bellini C (2008). & Van Wuytswinkel. Towards a systematic validation of references in real-time RT-PCR. The Plant Cell..

[26] Shi XQ (2013). Validation of reference genes for expression analysis by quantitative real-time PCR in *Leptinotarsa decemlineata* (Say). BMC Research Notes..

[27] Vandesompele, J *et al*. Accurate normalization of real-time quantitative RT-PCR data by geometric averaging of multiple internal control genes. *Genome biology*. **3**(7), 34-1 pp.research00 (2002). 10.1186/gb-2002-3-7-research0034 PMC126239 12184808

[28] Tan, Q.Q., Liu, W., Zhu, F., Lei, C.L. & Wang, X.P. Fatty acid synthase 2 contributes to diapause preparation in a beetle by regulating lipid accumulation and stress tolerance genes expression. *Scientific Reports*. **7**, p 40509 (2017). 10.1038/srep40509 PMC5223116 28071706

[29] Memarizadeh N, Zamani P, Sajedi RH, Ghadamyari M (2014). Purification and Characterization of Midgut α-Glucosidase from Larvae of the Rice Green Caterpillar, *Naranga aenescens* Moore. Journal of Agricultural Science and Technology..

[30] Bustin SA (2002). Quantification of mRNA using real-time reverse transcription PCR (RT-PCR): trends and problems. Journal of molecular endocrinology..

[31] Valasek MA, Repa JJ (2005). The power of real-time PCR. Advances in physiology education..

[32] de Jesus Miranda V (2013). Validation of reference genes aiming accurate normalization of qPCR data in soybean upon nematode parasitism and insect attack. BMC research notes..

[33] Zhang, S. *et al*. Identification and validation of reference genes for normalization of gene expression analysis using qRT-PCR in *Helicoverpa armigera* (Lepidoptera: Noctuidae). *Gene*. **555**(2), pp 393-402 (2015). 10.1016/j.gene.2014.11.038 25447918

[34] Ma KS (2016). Identification and Validation of Reference Genes for the Normalization of Gene Expression Data in qRT-PCR Analysis in *Aphis gossypii* (Hemiptera: Aphididae). Journal of Insect Science..

[35] Pfaffl MW, Tichopad A, Prgomet C, Neuvians TP (2004). Determination of stable housekeeping genes, differentially regulated target genes and sample integrity: BestKeeper–Excel-based tool using pair-wise correlations. Biotechnology letters..

[36] Migocka M, Papierniak A (2011). Identifcation of suitable reference genes for studying gene expression in cucumber plants subjected to abiotic stress and growth regulators. Mol. Breed..

[37] Silver N, Best S, Jiang J, Tein SL (2006). Selection of housekeeping genes for gene expression studies in human reticulocytes using real-time PCR. BMC Mol. Bio..

[38] Zheng YT, Li HB, Lu MX, Du YZ (2014). Evaluation and validation of reference genes for qRT-PCR normalization in *Frankliniella occidentalis* (Thysanoptera: Thripidae). PloS one..

[39] Chen F, Lu YY (2014). Selection of reference genes in *Phenacoccus solenopsis* (Hemiptera: Pseudococcidae) under heat stress. Acta Entomologica Sinica..

[40] Williams EA, Conzelmann M, Jékely G (2015). Myoinhibitory peptide regulates feeding in the marine annelid Platynereis. Frontiers in zoology..

[41] Yamanaka N (2010). Bombyx prothoracicostatic peptides activate the sex peptide receptor to regulate ecdysteroid biosynthesis. Proceedings of the National Academy of Sciences..

[42] Kim, Y. J. *et al*. MIPs are ancestral ligands for the sex peptide receptor. Proceedings of the *National Academy of Sciences*. **107**(14), pp 6520–6525 (2010). 10.1073/pnas.0914764107 PMC2851983 20308537

[43] Hua YJ (1999). Identification of a prothoracicostatic peptide in the larval brain of the silkworm, Bombyx mori. Journal of Biological Chemistry..

[44] Macours N, Hens K (2004). Zinc-metalloproteases in insects: ACE and ECE. Insect biochemistry and molecular biology..

[45] Livak KJ, Schmittgen TD (2001). Analysis of relative gene expression data using real-time quantitative PCR and the 2^−ΔΔCT^ method. Methods..

