# Supplementary material for: Reference genes validation in Phenacoccus solenopsis under various biotic and abiotic stress conditions
Source: Sci Rep. 2017 Oct 19;7:13520. doi: 10.1038/s41598-017-13925-9 (PMC5648885; doi:10.1038/s41598-017-13925-9)

**Supplementary Data**

**Reference genes validation in *Phenacoccus solenopsis* under various biotic and abiotic stress conditions**

**Authors:** Surjeet Kumar Arya^1, 2^, Gourav Jain^1^, Santosh Kumar Upadhyay^3^, Sarita^1,2^, Harpal Singh^1^, Sameer Dixit^1,2^, Praveen Chandra Verma^1, 2^*

^1^CSIR-National Botanical Research Institute, Council of Scientific and Industrial Research Rana Pratap Marg, Lucknow, UP, India.

^2^Academy of Scientific and Innovative Research (AcSIR), Anusandhan Bhawan, Room No: 310, 2-Rafi Marg, New Delhi, India.

^3^Department of Botany, Panjab University, Chandigarh-160014, India.

***** **Address for correspondence
Praveen Chandra Verma**

CSIR-National Botanical Research Institute,

Council of Scientific and Industrial Research

Rana Pratap Marg, Lucknow, UP, India.

Tel: +91-0522-2297922

Fax: +91-0522-2205836, 2205839

Email: praveencverma@nbri.res.in


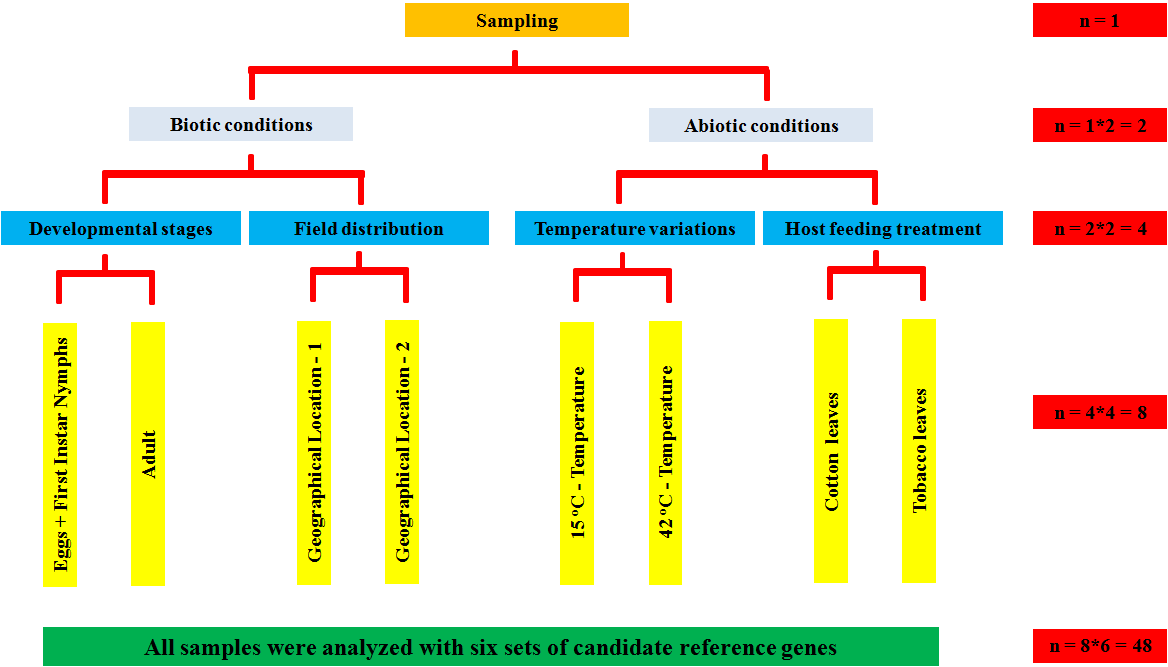


**Fig S1.** Flow chart representation of all the samples analyzed with six set of candidate reference genes under different biotic and abiotic conditions.

**
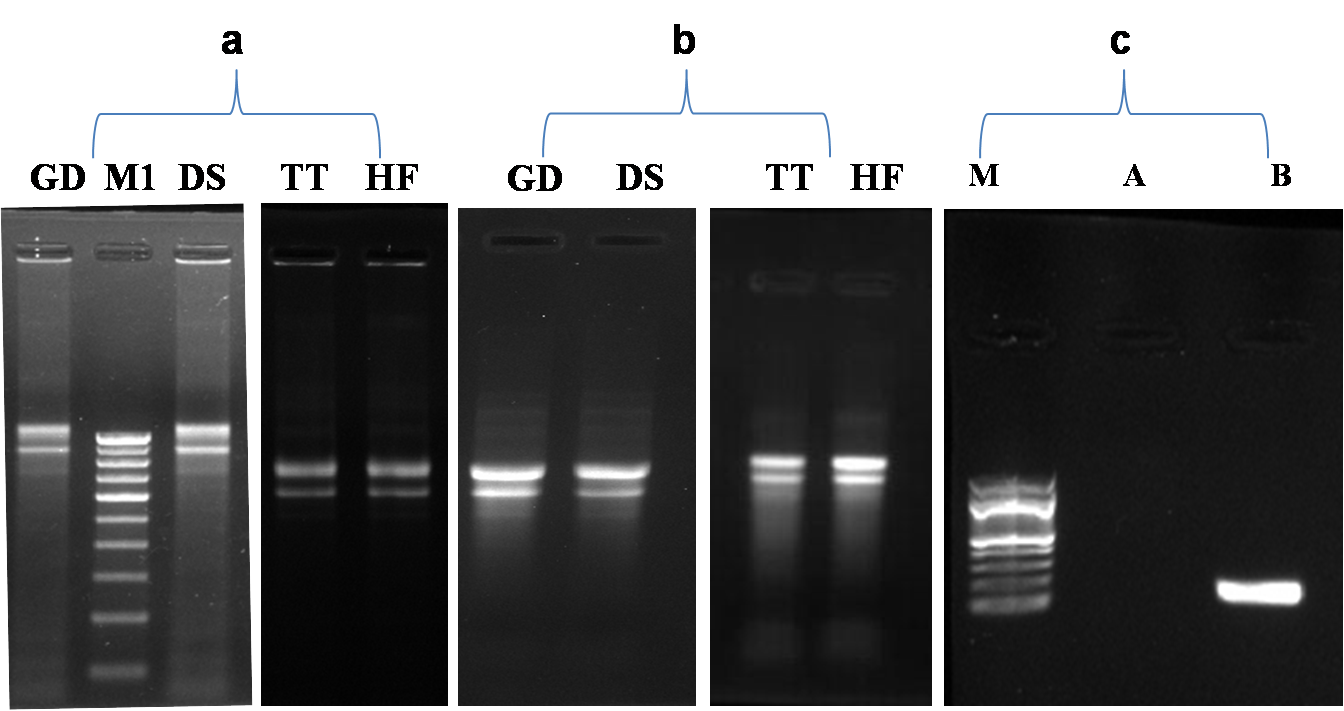
**

**
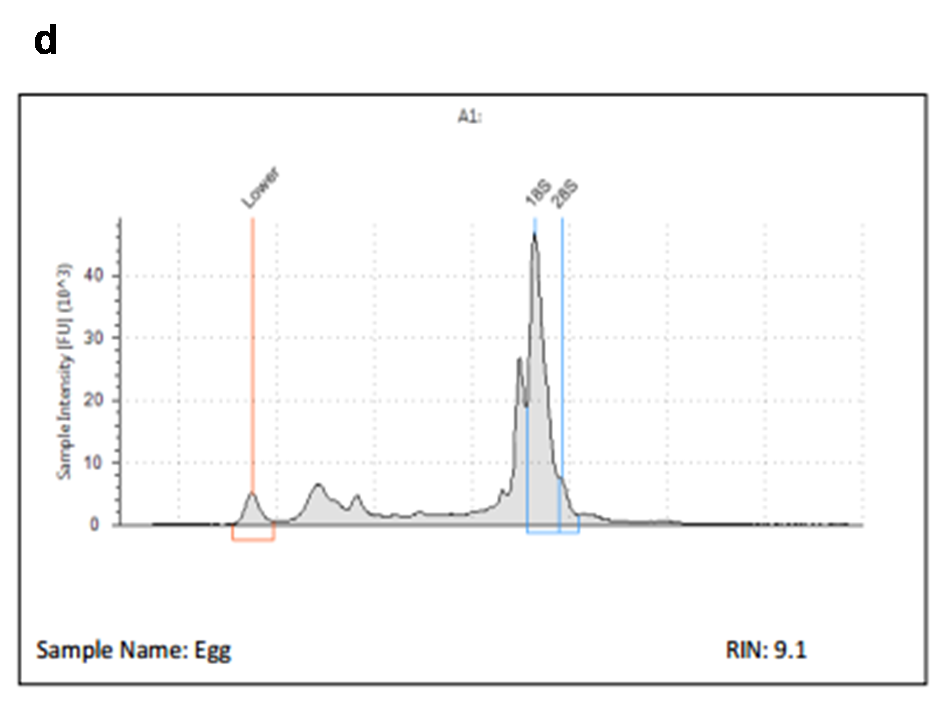
**

**GD:** Field distribution

**M1:** Marker 100 bp

**DS:** Developmental stage

**TT:** Temperature treatment

**HF:** Host feeding assay treatment

**M:** Marker bp

**RT:** PCR Checked with A (DNase Treated RNA) and B (cDNA template of DNase treated RNA) sample reaction

**Fig S2.** a) Gel image picture of RNA samples run at 1.2 % agarose gel RNA gel image before DNaseI treatment b) After DNAaseI treatment c) PCR check on A (DNase treated RNA) and B (cDNA template of DNaseI treated RNA), and d) Bio-analyzer check for RNA integrity, RIN value of RNA.

**
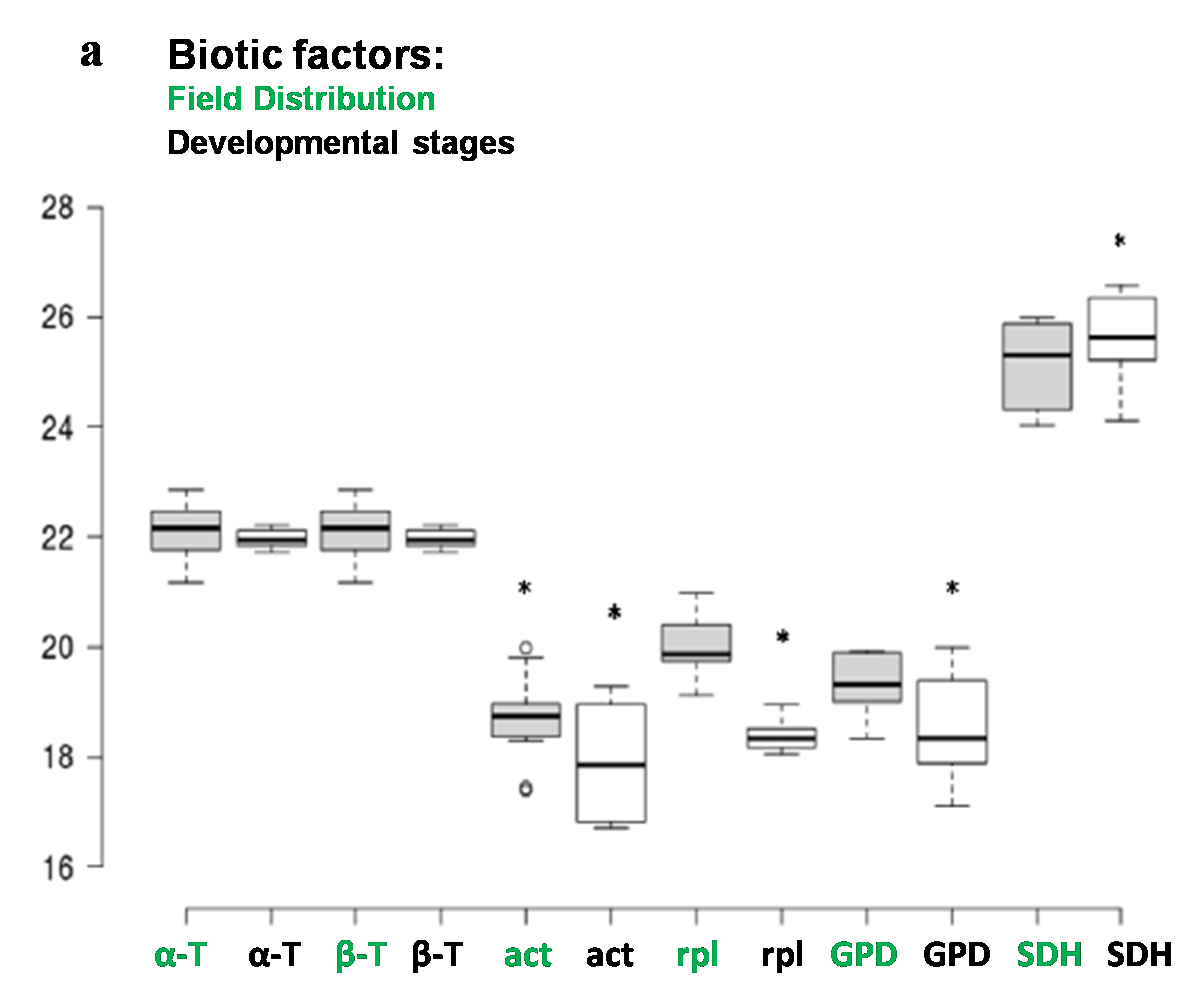
**

**
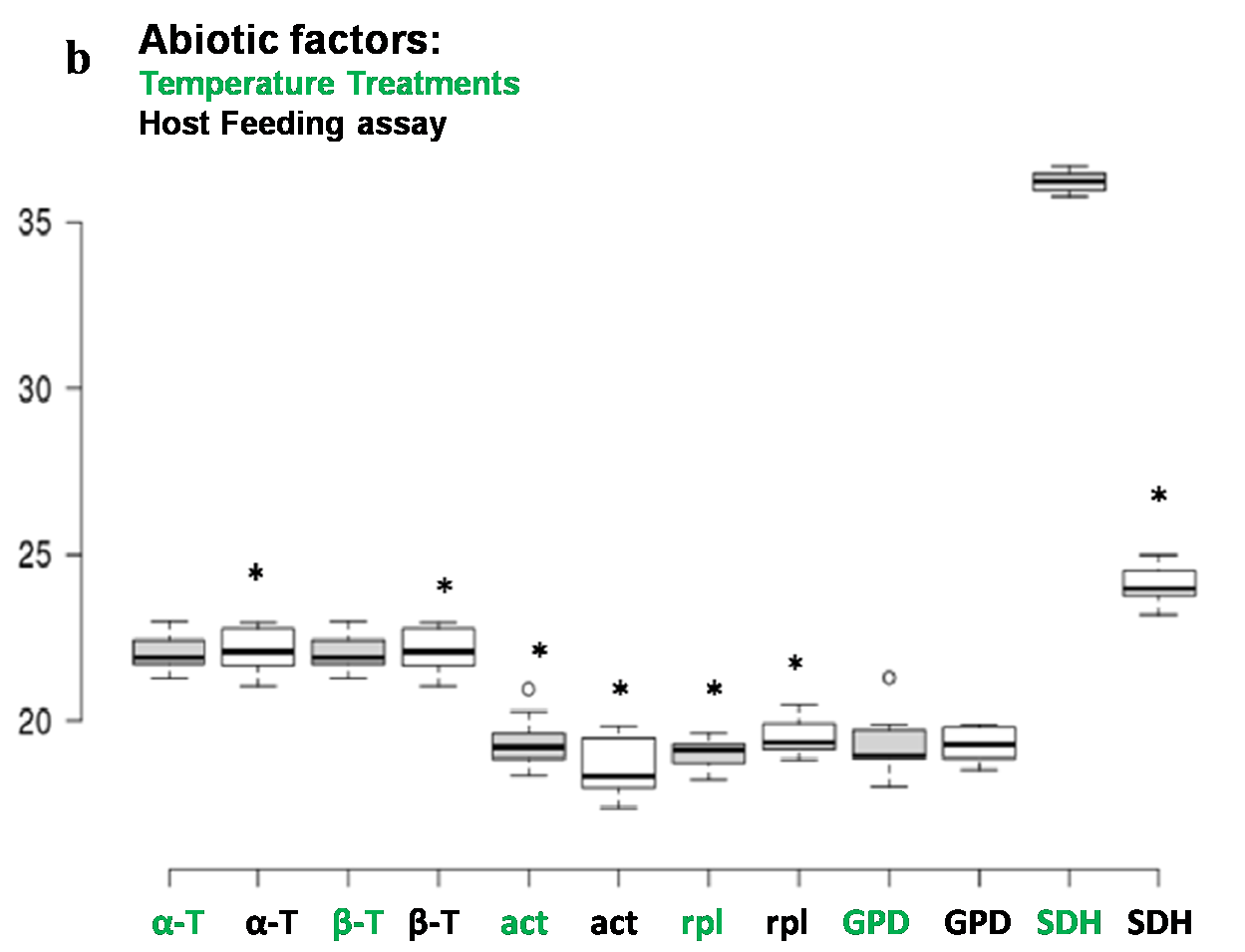
**

**Fig S3.** Comparison of Variance A) Two biotic treatment includes, Field distribution and Developmental stages, and B) Two Abiotic treatments include Temperature treatments and host-feeding assays. * Indicates p-value <0.05, (Mann-Whitney U Non-parametric test). Ct values are shown on Y-axis. Reference genes are shown on X-axis.

**Table S1.** Details of candidate reference genes, their primer sequences, product size and amplicon characteristics.

| **Gene Abbreviations** | **Primer sequence (forward/reverse primer)** | **Accession No.** | **Cellular Function** | **Amplicon Size** | **Tm (^o^C)** | **Slope** | **R^2^** | **Efficiency** |
| --- | --- | --- | --- | --- | --- | --- | --- | --- |
| ***ACT*** | 5’-CATCCTCCGTTTGGACTTGG-3’  5’-TCCAAGGCGACATAGCACAAT-3’ | KF384511 | Cytoskeleton structure  Protein | 144 bp | 75.97 | -3.332 | 0.979 | 1.995 |
| ***α-tublin*** | 5’-TACGTCGGTGAAGGTATGGAAGA-3’  5’-TCAACGGAGTCCATGCCAACT-3’ | KJ909508 | Cytoskeletal structural protein | 101 bp | 77.60 | -3.118 | 0.993 | 2.093 |
| ***β-tublin*** | 5’- CGCGAAGAATACCCCGATAGA-3’  5’- TACTGATAGGGTGGCGTTATATGGTT-3’ | KJ909511 | Cytoskeleton structure  protein | 102 bp | 77.97 | -3.182 | 0.996 | 2.062 |
| ***GAPDH*** | 5’- GTGGTGCCCAACAGAACATCA-3’  5’- TGGGACACGGAACGACATACC-3’ | KJ909509 | Glycolysis and  gluconeogenesis | 110 bp | 80.52 | -3.181 | 0.991 | 2.062 |
| ***SDH*** | 5’- GCGAATGCGTAGGCGTGATA-3’  5’- GCTCTACCGTAACCGCCAGTT-3’ | KM098145 | Energy metabolism | 100 bp | 73.97 | -3.315 | 0.975 | 2.002 |
| **rpl32** | 5’- TCGTGTTCGTAGGAGATTCAAAGG-3’  5’- GGACGAGGACCTTACGGAACTT-3’ | KJ909510 | Ribosomal structural constituent | 110 bp | 78.88 | -3.263 | 0.975 | 2.025 |

**Table S2.** Distribution of the Ct values of each candidate reference genes across the different Biotic and abiotic stress conditions in *Phenacoccus solenopsis*.

| **Genes** | **Field_Distrubution** | | **Developmental_Stages** | | **Temperature_Treatments** | | **Host_Feeding_Assays** | |
| --- | --- | --- | --- | --- | --- | --- | --- | --- |
|  | **G012** | **L12** | **E12** | **A12** | **C12** | **H12** | **CO12** | **T12** |
| *β-tubulin* | 15.85 ± 0.48 | 16.68 ± 0.19 | 18.47 ± 0.48 | 16.73 ± 0.48 | 35.15 ± 0.37 | 35.91 ± 0.25 | 16.07 ± 0.37 | 16.55 ± 0.32 |
| *rpl32* | 21.93 ± 0.46 | 22.21 ± 0.58 | 21.97 ± 0.07 | 21.95 ± 0.06 | 22.01 ± 0.37 | 22.07 ± 0.19 | 21.75 ± 0.37 | 22.47 ± 0.39 |
| *α-tubulin* | 19.15 ± 0.5 | 18.21 ± 0.27 | 16.85 ± 0.08 | 18.96 ± 0.08 | 18.86 ± 0.31 | 19.8 ± 0.60 | 19.3 ± 0.37 | 17.92 ± 0.16 |
| *SDH* | 19.82 ± 0.22 | 20.28 ± 0.63 | 18.17 ± 0.06 | 18.57 ± 0.20 | 18.74 ± 0.32 | 19.29 ± 0.19 | 19.92 ± 0.37 | 19.13 ± 0.21 |
| *ACT* | 19.54 ± 0.11 | 19.14 ± 0.37 | 17.74 ± 0.28 | 19.38 ± 0.07 | 19.16 ± 0.95 | 19.34 ± 0.47 | 19.64 ± 0.37 | 18.95 ± 0.18 |
| *GAPDH* | 24.97 ± 0.09 | 25.26 ± 0.75 | 26.26 ± 0.29 | 24.91 ± 0.41 | 36.2 ± 0.35 | 36.26 ± 0.29 | 24.06 ± 0.37 | 24.12 ± 0.31 |

- **GO1:** Glass House Populations
- **L12:** Net-House Populations
- **E12:** Pooled samples from egg and first instars Nymphs
- **A12:** Adult female mealybugs
- **C12:** Cold treatments (15^O^C)
- **H12:** Heat treatments (45^O^C)
- **C012:** Cotton leaves
- **T12:** Tobacco leaves

**Table S3. Expression stability values and rankings of 6 candidate reference genes calculated using geNorm.**

| **Field_Distrubution** | | **Developmental_Stages** | | **Temperature_Treatments** | | **Host_Feeding_Assays** | | **All_Samples** | |
| --- | --- | --- | --- | --- | --- | --- | --- | --- | --- |
| **Genes** | **M-value** | **Gene** | **Value** | **Genes** | **M-value** | **Genes** | **M-value** | **Genes** | **M-value** |
| *β-tubulin* | 0.381 | *β-tubulin* | 0.55 | *GAPDH* | 0.33 | *β-tubulin* | 0.34 | *α-tubulin* | 0.24 |
| *rpl32* | 0.42 | *rpl32* | 0.65 | *β-tubulin* | 0.34 | *α-tubulin* | 0.34 | *ACT* | 0.29 |
| *α-tubulin* | 0.55 | *α-tubulin* | 0.87 | *α-tubulin* | 0.49 | *SDH* | 0.46 | *SDH* | 0.36 |
| *SDH* | 0.65 | *SDH* | 0.94 | *rpl32* | 0.52 | *rpl32* | 0.68 | *rpl32* | 0.45 |
| *ACT* | 1.07 | *ACT* | 1.39 | *SDH* | 0.52 | *GAPDH* | 0.75 | *β-tubulin* | 0.47 |
| *GAPDH* | 1.18 | *GAPDH* | 1.48 | *ACT* | 0.61 | *ACT* | 1.03 | *GAPDH* | 0.76 |

**Table S4. Expression stability values and rankings of 6 candidate reference genes calculated using NormFinder.**

| **Field_Distrubution** | | **Developmental_Stages** | | **Temperature_Treatments** | | **Host_Feeding_Assays** | | **All_Samples** | |
| --- | --- | --- | --- | --- | --- | --- | --- | --- | --- |
| **Genes** | **Stability Value (SV)** | **Gene** | **Stability Value (SV)** | **Genes** | **Stability Value (SV)** | **Genes** | **Stability Value (SV)** | **Genes** | **Stability Value (SV)** |
| *rpl32* | 0.13 | *rpl32* | 0.16 | *rpl32* | 0.02 | *rpl32* | 0.24 | *rpl32* | 0.08 |
| *β-tubulin* | 0.17 | *β-tubulin* | 0.17 | *α-tubulin* | 0.08 | *α-tubulin* | 0.37 | *β-tubulin* | 0.16 |
| *α-tubulin* | 0.35 | *α-tubulin* | 0.70 | *SDH* | 0.19 | *SDH* | 0.42 | *SDH* | 0.21 |
| *SDH* | 0.43 | *GAPDH* | 0.77 | *GAPDH* | 0.21 | *β-tubulin* | 0.44 | *GAPDH* | 0.22 |
| *ACT* | 0.47 | *ACT* | 0.77 | *β-tubulin* | 0.22 | *GAPDH* | 0.56 | *α-tubulin* | 0.35 |
| *GAPDH* | 0.55 | *SDH* | 0.78 | *ACT* | 0.23 | *ACT* | 0.63 | *ACT* | 0.49 |

**Table S5. Expression stability values and rankings of 6 candidate reference genes calculated using Refinder.**

| **Field_Distrubution** | | **Developmental_Stages** | | **Temperature_Treatments** | | **Host_Feeding_Assays** | | **All_Samples** | |
| --- | --- | --- | --- | --- | --- | --- | --- | --- | --- |
| **Gene** | **Geomean Of Ranking Value** | **Gene** | **Geomean Of Ranking Value** | **Gene** | **Geomean Of Ranking Value** | **Gene** | **Geomean Of Ranking Value** | **Gene** | **Geomean Of Ranking Value** |
| *β-tubulin* | 1 | *β-tubulin* | 1.19 | *rpl32* | 1.19 | *GAPDH* | 1.19 | *rpl32* | 2 |
| *rpl32* | 1.86 | *rpl32* | 1.41 | *α-tubulin* | 2.28 | *rpl32* | 2 | *ACT* | 2 |
| *α-tubulin* | 3.22 | *SDH* | 3.46 | *SDH* | 2.63 | *α-tubulin* | 2.83 | *β-tubulin* | 2.06 |
| *GAPDH* | 3.76 | *GAPDH* | 3.66 | *β-tubulin* | 2.99 | *SDH* | 3 | *GAPDH* | 2.45 |
| *SDH* | 4.68 | *α-tubulin* | 4.73 | *ACT* | 4.73 | *β-tubulin* | 5 | *SDH* | 5 |
| *ACT* | 5.73 | *ACT* | 6 | *GAPDH* | 6 | *ACT* | 6 | *α-tubulin* | 6 |

**Table S6.** Details of objective reference genes, their primer sequences, product size and amplicon characteristics.

| **Gene Name** | **Primer sequence (Forward/reverse)** | **Amplicon size (bp)** | **Cellular function** | **Tm ^o^C** |
| --- | --- | --- | --- | --- |
| **Myoinhibitory peptide (*MIP*)** | 5’-CAAATGTTGACGAGTGCCG-3’  5’-TTCGCCTCGTGTGTTATTGC-3’ | 111 bp | Neuropeptides | 75 |
| **Zinc_Metalloprotease (*Zn_Mp*)** | 5’-GTAAGCAACCTCAATACCACCAATA-3’  5’-GAACCCTGAGGCACAACAAAA-3’ | 154 bp | Metalloprotease | 81 |
| ***Fatty acid synthase* (*Fas*)** | 5’- ACCACGTCACCCATACAGAGGATAC-3’  5’-GCCATTGAGAACCCATTCCG-3’ | 131 bp | stress tolerance | 80 |
| ***Alpha glucosidase*** | 5’- AACCACGGGTGTTCAATGCT-3’  5’-TGTCGGATTTCATTTCTATAGCTCC-3’ | 139 bp | metabolism | 78 |

**Supplementary data 2:** PCR Product sequencing result showing the amplicon sequences of the given primers

1. **Actin**

**FORWARD PRIMER:** CATCCTCCGTTTGGACTTGG

**REVERSE PRIMER:** TCCAAGGCGACATAGCACAAT

>actin

CATCCTCCGTTTGGACTTGGCTGGTCGTGACTTAACTGACTACTTGATGAAAATCCTTACTGAACGTGGTTACAGTTTCACCACCACTGCTGAACGAGAAATCGTCCGTGATATCAAAGAAAAATTGTGCTATGTCGCCTTGGA

**
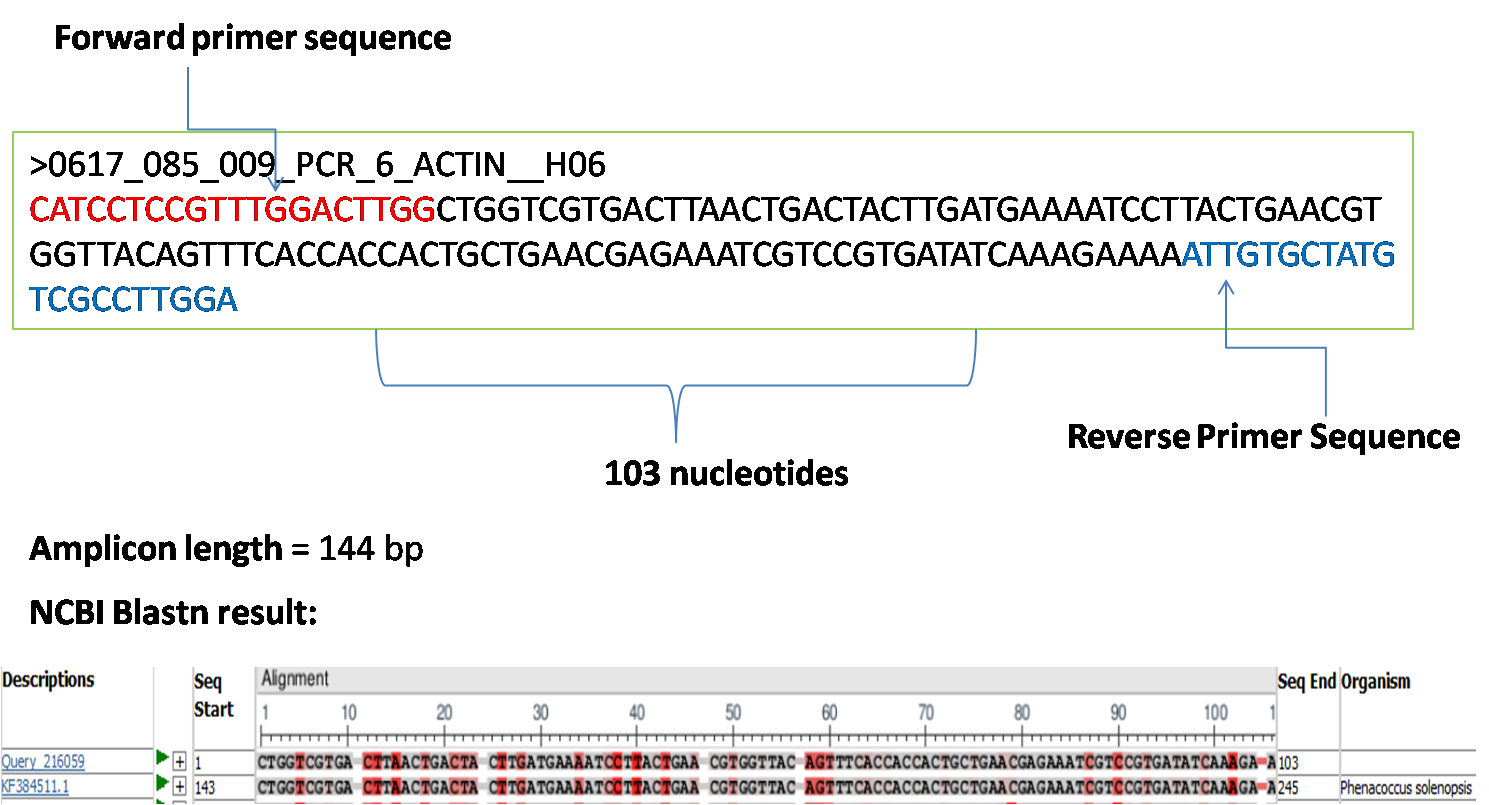
**

1. **α-tubulin**

**FORWARD PRIMER:** TACGTCGGTGAAGGTATGGAAGA

**REVERSE PRIMER:** TCCAAGGCGACATAGCACAAT

>α-tubulin

TACGTCGGTGAAGGTATGGAAGAAGGTGAATTCTCCGAAGCTCGTGAAGATTTAGCTGCTTTAGAGAAAGATTATGAAGAAGTTGGCATGGACTCCGTTGA

**
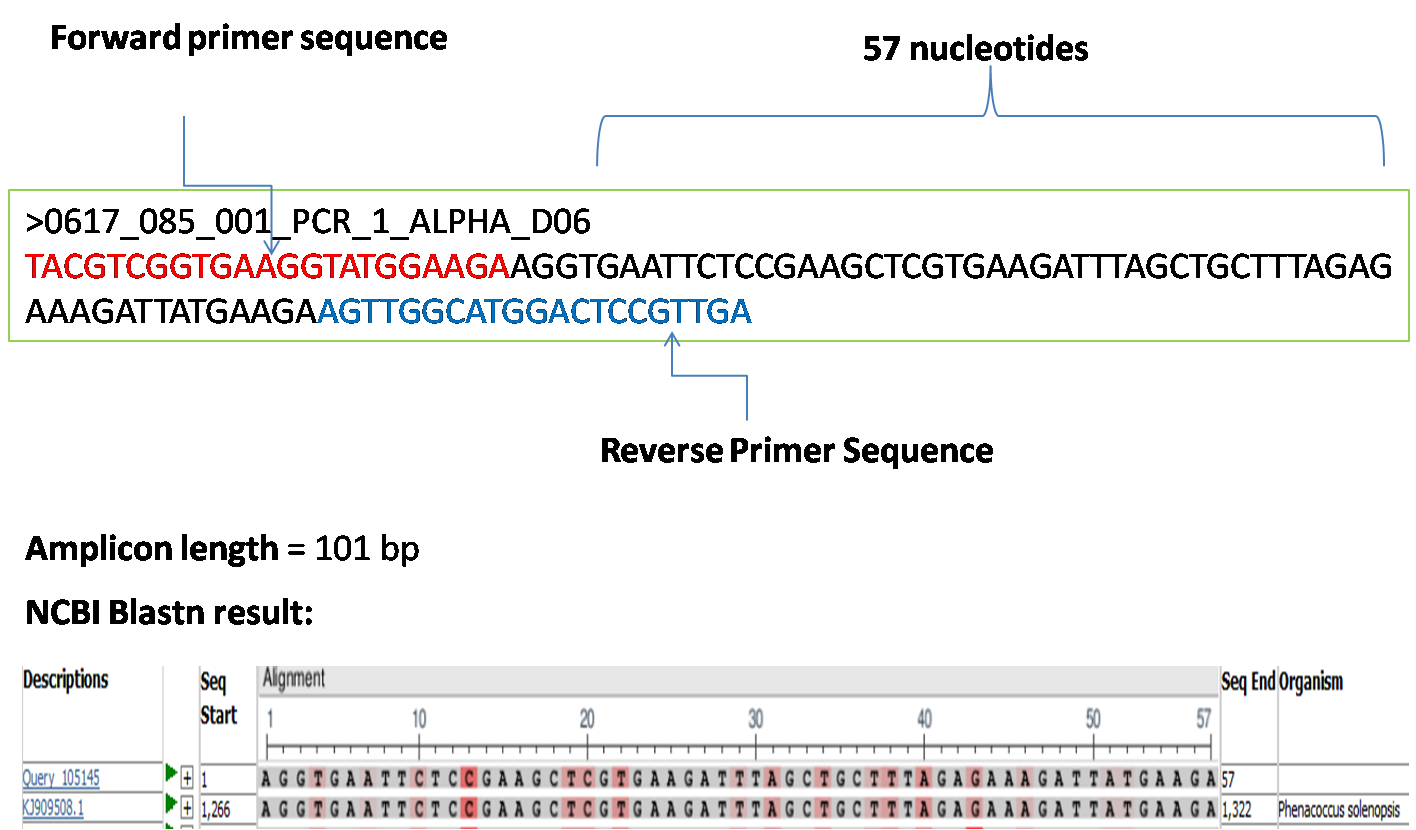
**

1. **β-tubulin**

**FORWARD PRIMER:** CGCGAAGAATACCCCGATAGA

**REVERSE PRIMER**: TACTGATAGGGTGGCGTTATATGGTT

>β-tubulin

CGCGAAGAATACCCCGATAGAATAATGAACACATACTCCGTTGTACCATCTCCCAAAGTTTCCGACACCGTAGTAGAACCATATAACGCCACCCTATCAGTA

**
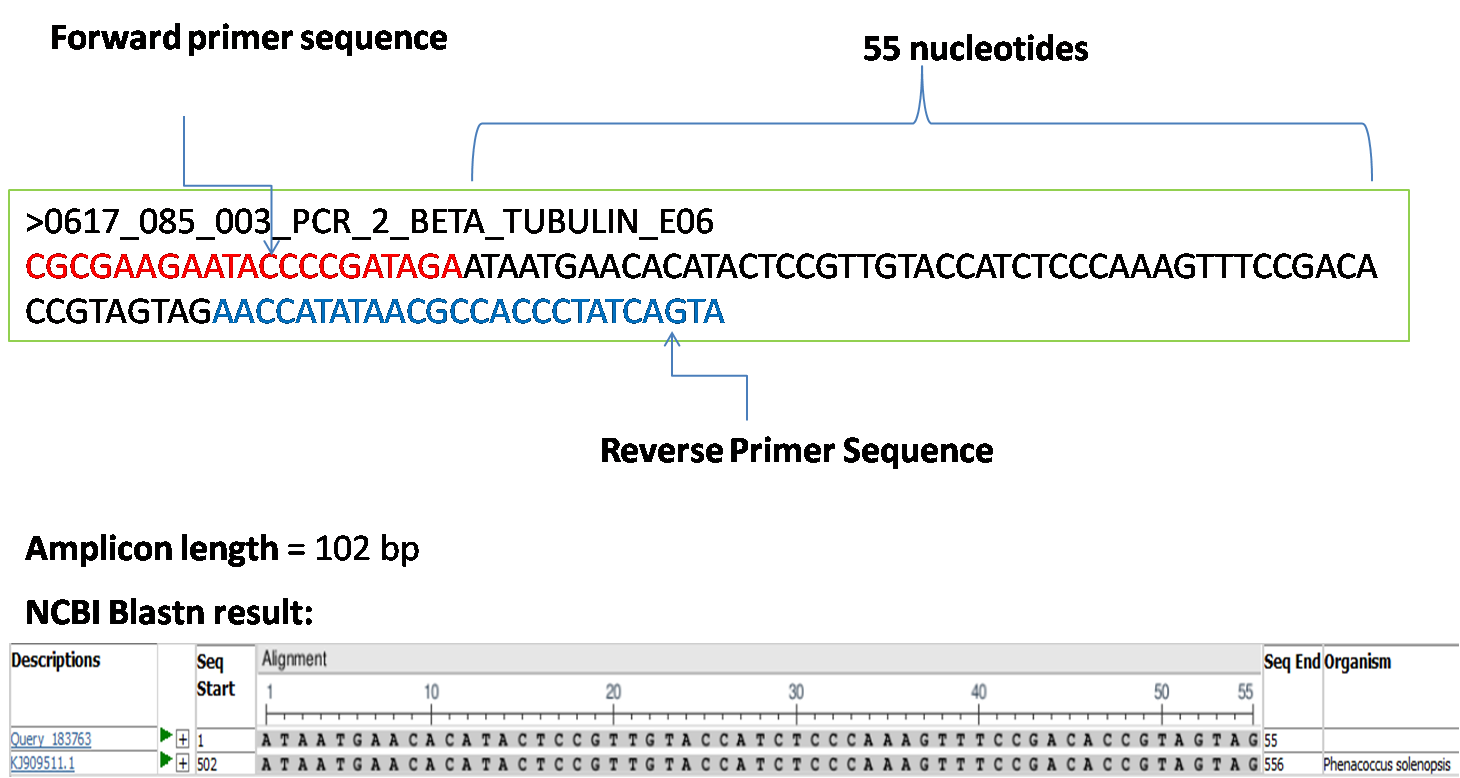
**

1. **GAPDH**

**FORWARD PRIMER:** GTGGTGCCCAACAGAACATCA

**REVERSE PRIMER:** TGGGACACGGAACGACATACC

>GAPDH

GTGGTGCCCAACAGAACATCATCCCAGCATCGACTGGTGCTGCCAAAGCTGTCGGTAAAGTTATTCCTTCTTTGAATGGTAAACTTACCGGTATGTCGTTCCGTGTCCCA

**
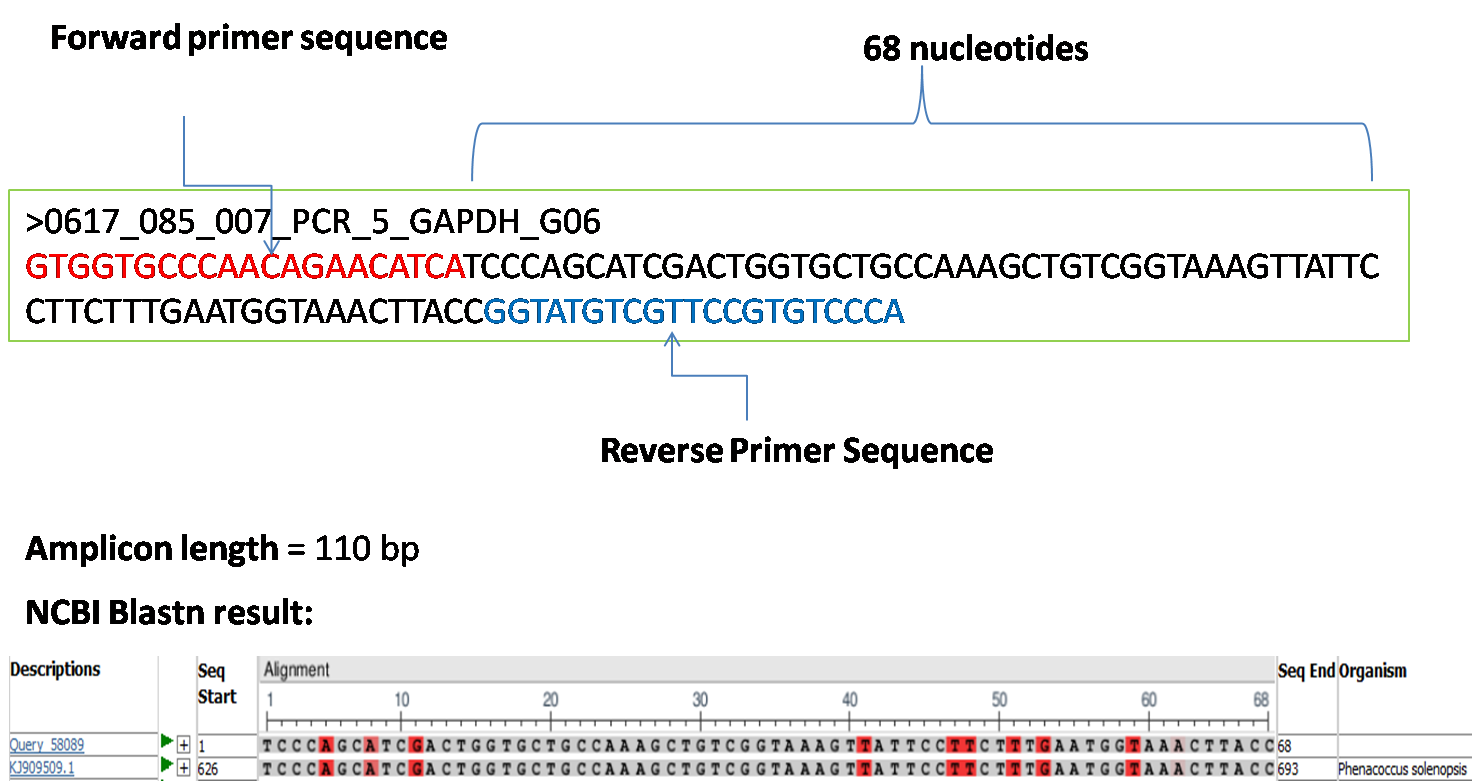
**

1. **Ribosomal protein 32**

**FORWARD PRIMER:** TCGTGTTCGTAGGAGATTCAAAGG

**REVERSE PRIMER:** GGACGAGGACCTTACGGAACTT

**> rpl 32**

TCGTGTTCGTAGGAGATTCAAAGGCCAATATTTAATGCCAAATGTTGGTTATGGAAGTAATAAAAAAACTAAACATATGCTTCCCAATAAGTTCCGTAAGGTCCTCGTCC

**
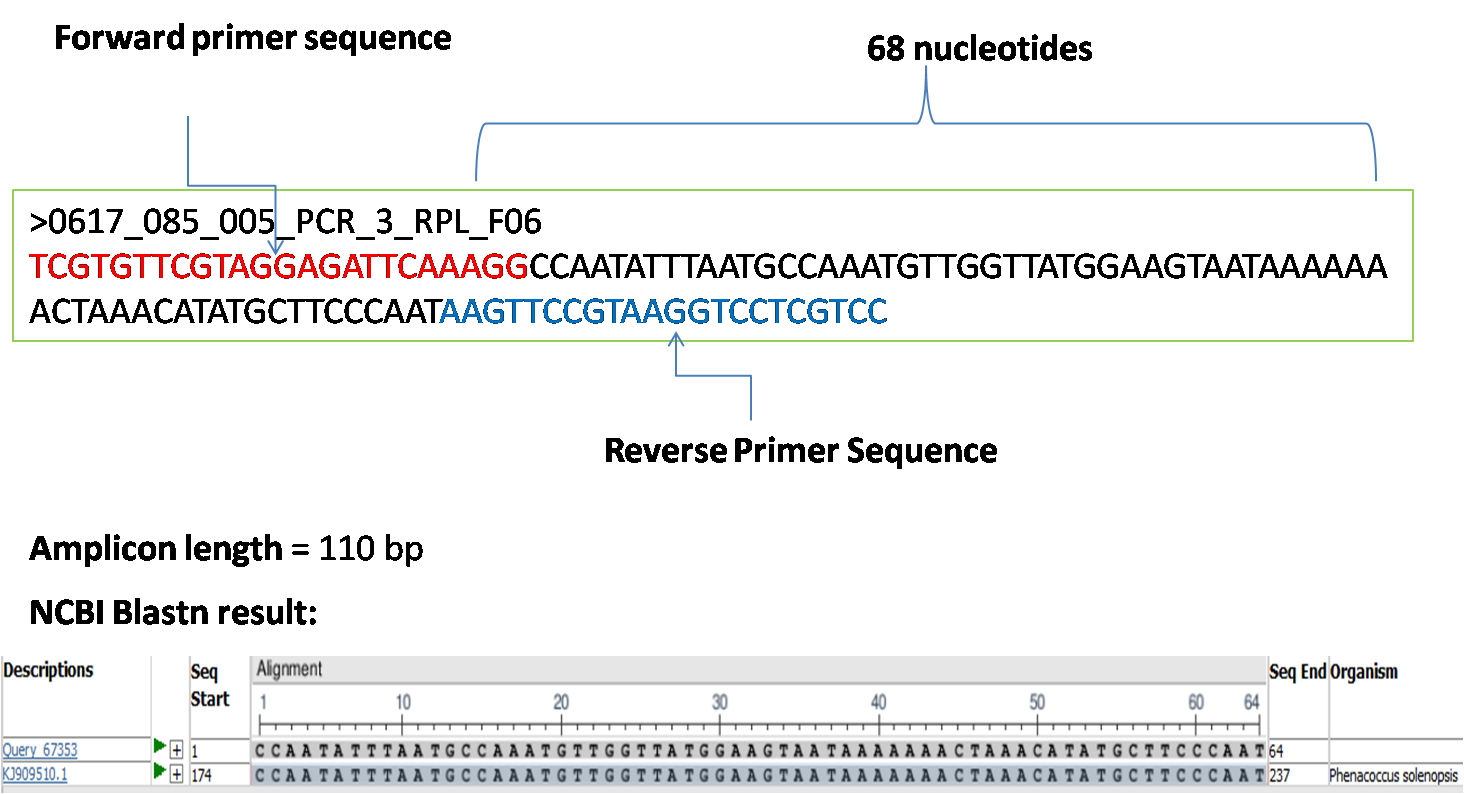
**

1. **Succinate dehydrogenase**

**FORWARD PRIMER:** GCGAATGCGTAGGCGTGATA

**REVERSE PRIMER:** GCTCTACCGTAACCGCCAGTT

> **SDH**

GCGAATGCGTAGGCGTGATAGCCTTATGCCTGGAAGATGGCAGCATTCATCGTTTTAAAGCAAAAAACACGATTTTAGCAACTGGCGGTTACGGTAGAGC


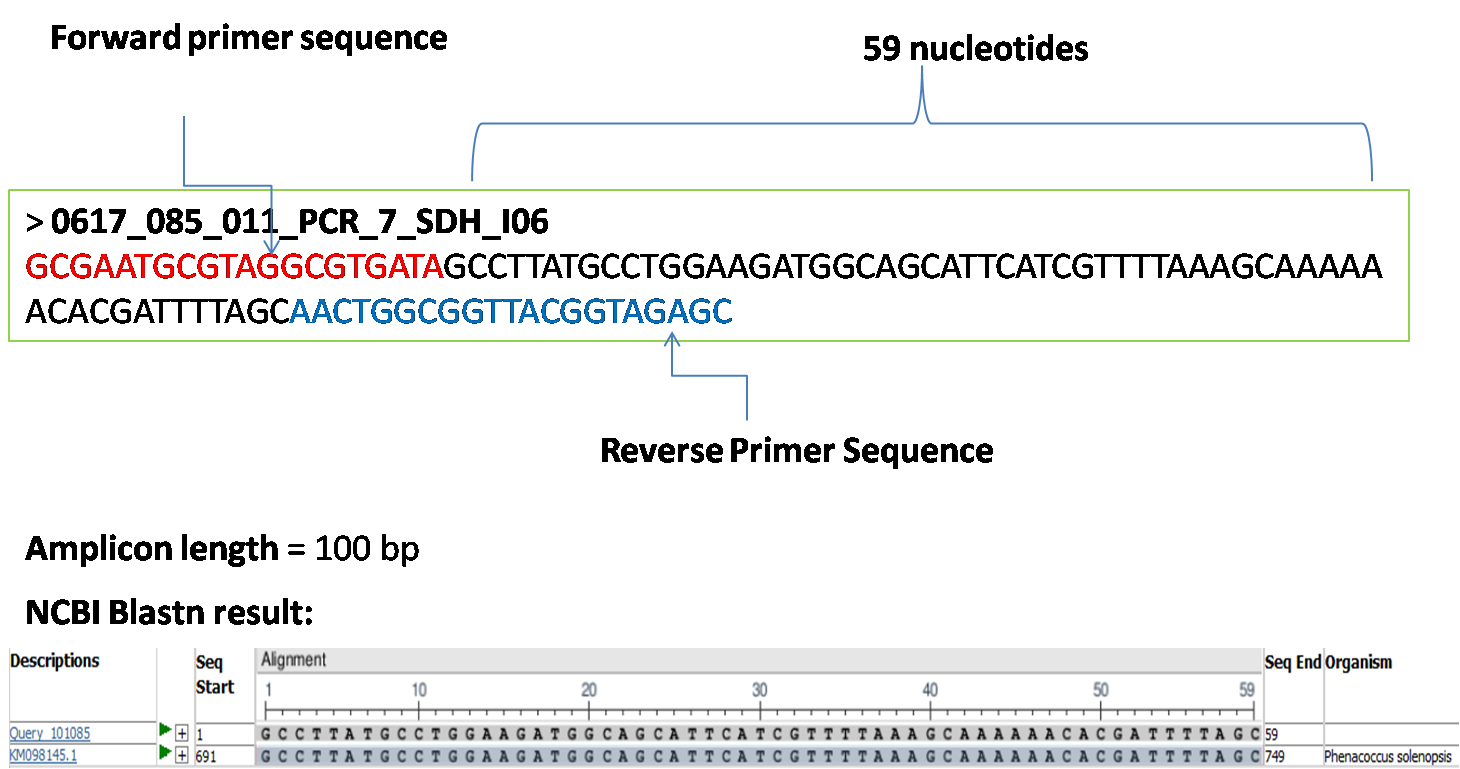


1. **Myoinhibitory peptide**

**FORWARD PRIMER:** CAAATGTTGACGAGTGCCG

**REVERSE PRIMER:** TTCGCCTCGTGTGTTATTGC

**>myoinhibitory_peptide**

CAAATGTTGACGAGTGCCGATATTTGTGTTGGTGTTAGTGTTGGCATTGGAATTCGAACTGGCGATATTGGACGAGCTTTCGCTTTTTGCGGCAATAACACACGAGGCGAA

**
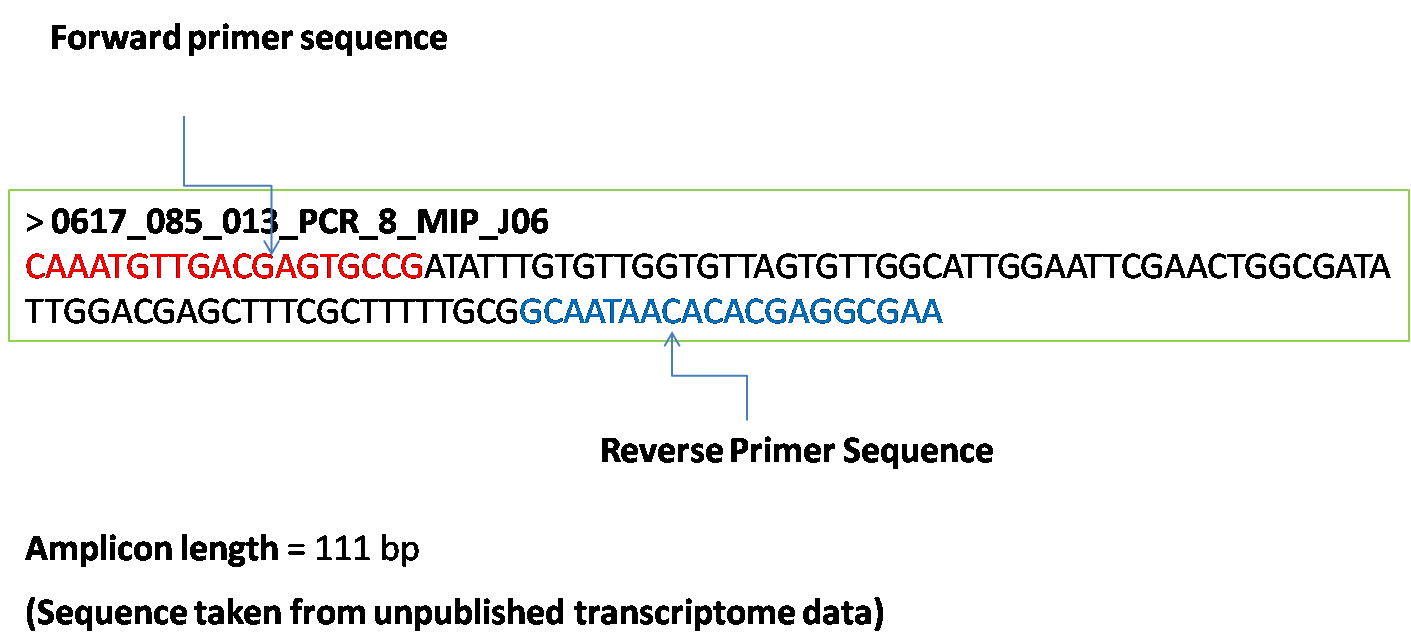
**

1. **Zinc metalloprotease**

**FORWARD PRIMER:** GTAAGCAACCTCAATACCACCAATA

**REVERSE PRIMER:** GAACCCTGAGGCACAACAAAA

>Zinc_metalloprotease

GTAAGCAACCTCAATACCACCAATATCAGCGAAATTCTCTGATGAAGTTTGCAGACCATTAACCTTGTTTCCAGTTTCTAAATTTGTGAAAGTAGAATATTGTTGCGCAATACATTCGAAAGTTTGAAAATGTTTTTGTTGTGCCTCAGGGTTC

**
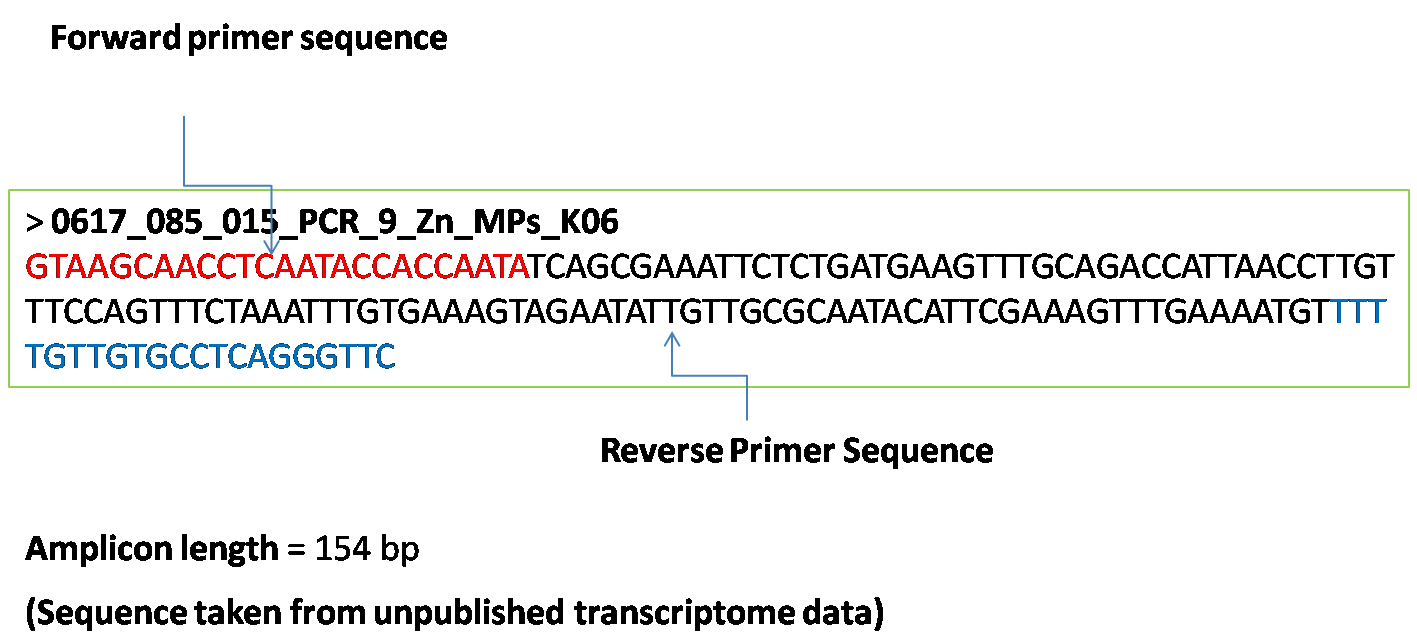
**

1. **Fatty acid synthase**

**FORWARD PRIMER:** ACCACGTCACCCATACAGAGGATAC

**REVERSE PRIMER:** CGGAATGGGTTCTCAATGGC

>Fatty acid synthase

ACCACGTCACCCATACAGAGGATACGTCATATTTAATAAAAACTTCAGCATCAAAAGTCATCATGTTCAGCGTATTAAAGAAAACTCTCCTCAACTTTGGTTCATCTATCCCGGAATGGGTTCTCAATGGC


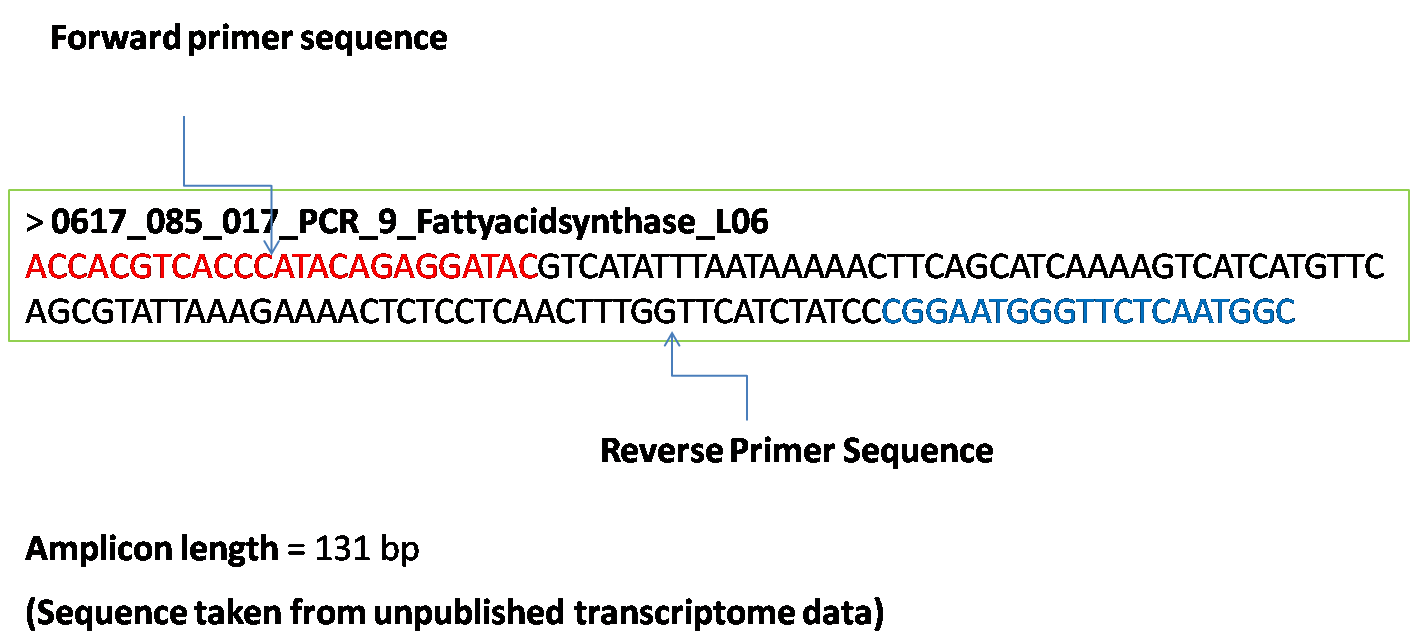


1. **Alpha_glucosidase**

**FORWARD PRIMER:** ACCACGTCACCCATACAGAGGATAC

**REVERSE PRIMER:** CGGAATGGGTTCTCAATGGC

>Alpha_glucosidase

AACCACGGGTGTTCAATGCTGCTGTGATTAATTACCATATCGATTACAAGTTTTAAGCCTCGAGCTTTTATTCCTTCTACAAGCTTCTCAAAATCTTCCATGGTACCAAACTGCGGAGCTATAGAAATGAAATCCGACA


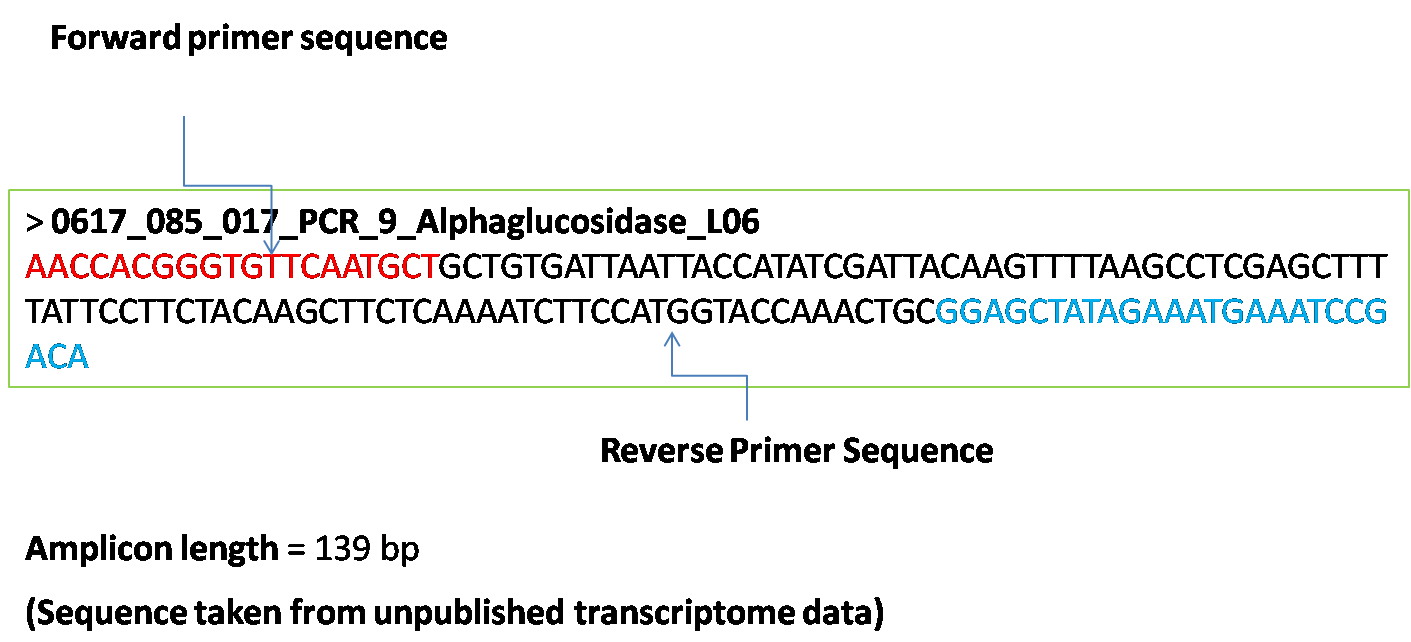

Supplement: Supplementary file 1 — Supplementary File [file 41598_2017_13925_MOESM1_ESM.doc]
